# Optimization of Pressurized Liquid Extraction (PLE) Parameters for Extraction of Bioactive Compounds from *Moringa oleifera* Leaves and Bioactivity Assessment

**DOI:** 10.3390/ijms25094628

**Published:** 2024-04-24

**Authors:** Theodoros Chatzimitakos, Vassilis Athanasiadis, Konstantina Kotsou, Martha Mantiniotou, Dimitrios Kalompatsios, Ioannis Makrygiannis, Eleni Bozinou, Stavros I. Lalas

**Affiliations:** Department of Food Science and Nutrition, University of Thessaly, Terma N. Temponera Street, 43100 Karditsa, Greece; tchatzimitakos@uth.gr (T.C.); kkotsou@agr.uth.gr (K.K.); mmantiniotou@uth.gr (M.M.); dkalompatsios@uth.gr (D.K.); ioanmakr1@uth.gr (I.M.); empozinou@uth.gr (E.B.); slalas@uth.gr (S.I.L.)

**Keywords:** Moringa, pressurized liquid extraction, polyphenols, flavonoids, antioxidants, response surface methodology, Box–Behnken design, Bayes plot analysis, multiple factor analysis, partial least squares analysis

## Abstract

*Moringa oleifera* leaves are rich sources of bioactive compounds with potential health benefits, including antioxidants and anti-inflammatory agents. Pressurized liquid extraction (PLE) stands out as a promising technique for effectively extracting valuable compounds from natural sources. In this study, we aimed to optimize PLE parameters, such as temperature, extraction duration, and pressure, to maximize bioactive compound (polyphenols, flavonoids, and ascorbic acid) yield from *M. oleifera* leaves and evaluate their antioxidant and anti-inflammatory activities. According to the outcomes of this research, the maximum achieved total polyphenol content was 24.10 mg gallic acid equivalents (GAE)/g of dry weight (dw), and the total flavonoid content was increased up to 19.89 mg rutin equivalents (RtE)/g dw. Moreover, after HPLC-DAD analysis, neochlorogenic and chlorogenic acids, catechin and epicatechin, rutin, and narirutin were identified and quantified. As far as the optimum ascorbic acid content is concerned, it was found to be 4.77 mg/g dw. The antioxidant activity was evaluated by three different methods: ferric reducing antioxidant power (FRAP), the DPPH method, and the anti-hydrogen peroxide activity (AHPA) method, resulting in 124.29 μmol ascorbic acid equivalent (AAE)/g dw, 131.28 μmol AAE/g dw, and 229.38 μmol AAE/g dw values, respectively. Lastly, the albumin denaturation inhibition was found to be 37.54%. These findings underscore the potential of PLE as an efficient extraction method for preparing extracts from *M. oleifera* leaves with the maximum content of bioactive compounds.

## 1. Introduction

The most common species of the Moringaceae family, renowned for its significant pharmaceutical and therapeutic properties, is *Moringa oleifera* (MO) [1], primarily cultivated in subtropical and tropical regions across many countries worldwide [2]. The MO tree is grown for its edible leaves, flowers, and nutritious pods [3], with its leaves attracting the attention and interest of scientists globally [4]. The leaves are highly esteemed for their abundant content of polyphenolic compounds, primarily flavonoids, and phenolic acids [5], including quercetin, kaempferol, myricetin, chlorogenic acid, and rutin [6]. Furthermore, the leaves are a good source of numerous vitamins present in MO leaves, such as vitamin C, vitamin E, and β-carotene [7,8], while they contain compounds that exhibit antioxidant, anti-inflammatory, anticancer, and cardioprotective properties [2]. Their richness in nutrients combined with their overall beneficial attributes make them a valuable addition to human nutrition [9]. Additionally, MO leaves serve as supplementary feed for livestock [10], poultry [11], and edible insects [12] due to their high nutritional value.

Due to the popularity of MO leaves in the scientific community, numerous studies have been conducted to examine various extraction methods (conventional and green) to ensure the highest quantity of phenolic compounds, the primary bioactive elements contained in MO leaves [13,14,15,16,17,18,19]. Regarding conventional extraction methods, simple stirring with deionized water at 80 °C for 120 min has been performed, and the total amount of flavonoids has been determined to be 31.28 mg quercetin equivalent (QE)/g [14]. Additionally, the Soxhlet method has been employed with various solvents, and the quantity of total polyphenols has been determined. Specifically, using 50% ethanol, 70% ethanol, and water for 20 h, the total polyphenol content (TPC) of 132.5 mg chlorogenic acid equivalents (CGA)/g [15], 124.70 mg CGA/g [15], and 45.81 mg gallic acid (GAE)/g was recorded [16], respectively. Lastly, the same ethanolic solvents were also utilized for extraction through maceration at room temperature for ~72 h, resulting in a TPC of 72.2 mg CGA/g with 50% ethanol and 132.30 mg CGA/g with 70% ethanol [15].

In addition to the application of conventional extraction methods, there are numerous examples of using green extraction methods to study the quantity of polyphenols contained in MO leaves [17,18,19]. Characteristic examples include ultrasound-assisted extraction, supercritical fluid extraction, microwave-assisted extraction, and pulsed electric fields. The use of ultrasound-assisted extraction was conducted for 15 min at room temperature with 50% ethanol, resulting in 47.00 mg GAE/g TPC [17]. Furthermore, supercritical fluid extraction was performed using two different solvents: carbon dioxide-expanded ethanol (50–200 °C) and pressurized hot water (50–70 °C) for 280 min, recording values of 20.30 and 62.40 mg GAE/g TPC, respectively [18]. Moreover, when microwave treatment (350 W) was evaluated for 2 min at 80 °C, the TPC in MO leaves was 36.59 mg GAE/g [19]. Last but not least, in a previous study, extraction using the pulsed electric field method (time: 40 min, room temperature, pulse duration (PD): 20 ms, and pulse interval (PI): 100 μs) ensured a value of 40.24 mg GAE/g TPC [19].

As it is known, green extraction methods represent innovative techniques in extraction, either used alone or in combination with conventional methods. These methods have piqued the interest of researchers due to their ability to effectively isolate a wide array of bioactive substances [20]. In addition to the previously mentioned green extraction techniques, there is another method that requires further exploration, known as pressurized liquid extraction (PLE) [21]. PLE is performed by applying heat and high pressure to the solvent and solid extraction sample in order to increase the solubility, mass transfer rate, and extractability of the sample, improving the speed and extraction efficiency [22]. It also keeps the solvents close to their supercritical range, where they provide increased extraction properties [23]. Thus, PLE is recognized as a high-performance green extraction method for sustainably extracting bioactive compounds from natural sources, such as plants, to produce extracts with various applications in the food industry [24].

Bearing in mind the rich nutritional value of MO leaves along with the plethora of therapeutic properties they offer, the necessity for their multifaceted study to find the most ideal extraction methods and parameters for ensuring the maximum amount of these nutrients and antioxidants is evident. Also, given the efficiency offered by PLE in the isolation of bioactive compounds from plants and the experimental shortcomings in the extraction of MO leaves by this innovative technique, the objective of this study is to investigate and document the optimal extraction parameters for MO leaves using PLE, intending to achieve maximum yields of polyphenols, antioxidants, and anti-inflammatory agents.

## 2. Results and Discussion

### 2.1. Optimization of Extraction Parameters

In order to enhance extraction efficiency, it is imperative to optimize the extraction parameters. The extraction process may be multifaceted due to the presence of various bioactive compounds, which can lead to variations in solubility and polarity [25]. Moreover, the extraction technique and diverse processing parameters exert a significant influence on the extract yield and antioxidant capacity. Hence, it is vital to increase the performance of this process [26]. Recently, there has been remarkable growth in the development of extraction methods that reduce the reliance on harmful and toxic solvents, preserve human well-being, and require minimal energy. The incorporation of an environmentally sustainable solvent is vital for the efficient execution of this methodology [27]. Water is a readily accessible and sustainable solvent due to its exceptional efficacy in extracting polar compounds, cost-effectiveness, and non-toxicity towards humans [27]. Considering all these, and to achieve maximum recovery of bioactive compounds, various parameters such as extraction temperature and duration, pressure, and liquid-to-solid ratio were examined. These parameters were examined in combination, as shown in Table 1. Table 1 also shows the values of TPC, total flavonoid content (TFC), ferric reducing antioxidant power (FRAP), 2,2-diphenyl-1-picrylhydrazyl (DPPH), and anti-hydrogen peroxide activity (AHPA), which were used as criteria to optimize the extraction process. Additional parameters evaluated for optimization were ascorbic acid content (AAC) and albumin denaturation inhibition (ADI), which are shown in Table 2 along with the color analysis of each extract.

According to the results displayed in Table 1, it can be remarked that the liquid-to-solid ratio impacts the extraction performance, since when the ratio increases, the recovery of bioactive compounds increases too. Regarding pressure, lower values do not seem to favor the recovery of bioactive compounds, while higher values appear to increase the recovery, suggesting that the combination of high temperature and high-pressure conditions can significantly diminish the strong interaction between the solute and the matrix, which arises from van der Waals forces or hydrogen bonds, as well as the dipole attraction between solute molecules and the active sites of the sample matrix [28]. This enhances the efficiency of extracting solute molecules, decreases the energy required for analysis, and lowers the viscosity of the solvent. Consequently, it reduces the solvent resistance to the sample matrix and facilitates its diffusion into the sample [28]. The color analyses in Table 2 indicate that the higher the ratio, the lighter the color of the extracts. Also, it can be observed that intermediate pressure values lead to high albumin denaturation inhibition of the extracts, which could imply a possible anti-inflammatory activity of the extracts, while the ascorbic acid content is not considerably affected by the pressure value at which the extraction takes place.

**Figure 1:**
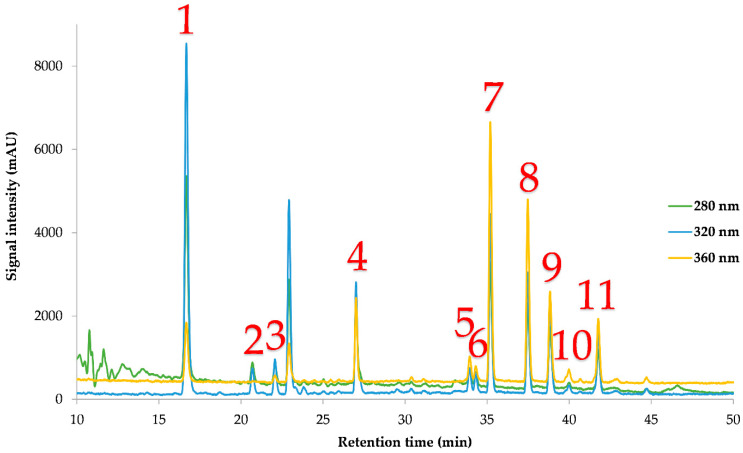
Representative HPLC chromatogram at 280, 320, and 360 nm of *M. oleifera* extract demonstrating polyphenols identified. 1: neochlorogenic acid; 2: catechin; 3: chlorogenic acid; 4: epicatechin; 5: ferulic acid; 6: rutin; 7: quercetin 3-β-*D*-glucoside; 8: narirutin; 9: kaempferol-3-glucoside; 10: apigenin-7-*O*-glucoside; 11: myricetin.

Table 3 shows the concentrations of the individual polyphenols determined by HPLC-DAD from each analysis under different PLE conditions, and Figure 1 shows a representative chromatogram of an MO extract. Based on the findings presented in Table 3, it can be inferred that neochlorogenic acid is the main compound in the MO PLE extracts, followed by quercetin-3-β-*D*-glucoside and myricetin and kaempferol-3-glucoside. These results are in line with other studies [6,29], in which these polyphenols have been detected, too. More specifically, Nouman et al. [29] also reported neochlorogenic acid (3-caffeoylquinic acid) to be the main compound in MO leaf extracts through liquid chromatography-mass spectrometry, followed by kaempferol-3-glucoside. As can be seen in Figure 1, there are some unidentified peaks. According to the literature, the unidentified peak at ~23 min could possibly be attributed to the presence of cryptochlorogenic acid, as the absorbance spectrum is similar to that of the unknown compound, which is known to exist in MO leaves in sufficient amounts [30,31,32]. In Figure S1, the UV spectra of the unknown peak are depicted, and they match the UV spectra for cryptochlorogenic acid, provided in an earlier study [32], strengthening our tentative identification.

Table S1 displays the statistical parameters, second-order polynomial equations (models), and coefficients (coefficients > 0.92) derived for each model, indicating a strong match for the developed models. Figures S2–S6 provide plots comparing the actual response to the projected response for each parameter under examination, along with the desirability functions. Figures S7–S11 exhibit three-dimensional response plots for all responses under investigation.

Taking into consideration Figure S2, it is apparent that there is an excellent fit of the developed model to the responses of the TPC of the extracts, with the desirability function being ~0.99. In Figure S3, it can be observed that the developed models predicting the responses of the TFC have a good fit, too. The same conclusion can be drawn by observing Figures S4–S6, which refer to the antioxidant capacity of the extracts through DPPH assay, FRAP assay, and AHPA assay, respectively, demonstrating high desirability functions, too. Figures S7–S11 point out that higher values of pressure, temperature, and ratio were considerably effective for achieving the maximum yield, even at short extraction durations, across all parameters examined (TPC, TFC, FRAP, DPPH, and AHPA). Optimizing extraction length and temperature is crucial for minimizing the consumption of energy in the extraction process. Given the verified efficacy of both brief and extended extraction periods in previous studies, an in-depth investigation is necessary to assess the impact of time on extraction [27]. Elevated temperatures assist in improving extraction processes by increasing the solubility of solutes and increasing diffusion coefficients [33]. It has been reported that the application of water as an extraction solvent is enhanced by the significant alterations in its physical–chemical properties, particularly in its dielectric constant (*ε*), when subjected to elevated temperatures and pressures [34]. This is attributed to the fact that the dielectric constant serves as an indicator of the polarity of the solvent and plays a pivotal role in defining the interactions between solutes and solvents. In the case of water, raising the temperature under moderate pressure can lead to a substantial reduction in this constant [35].

### 2.2. Impact of Extraction Parameters to Responses through Bayes Plot Analysis

**Figure 2:**
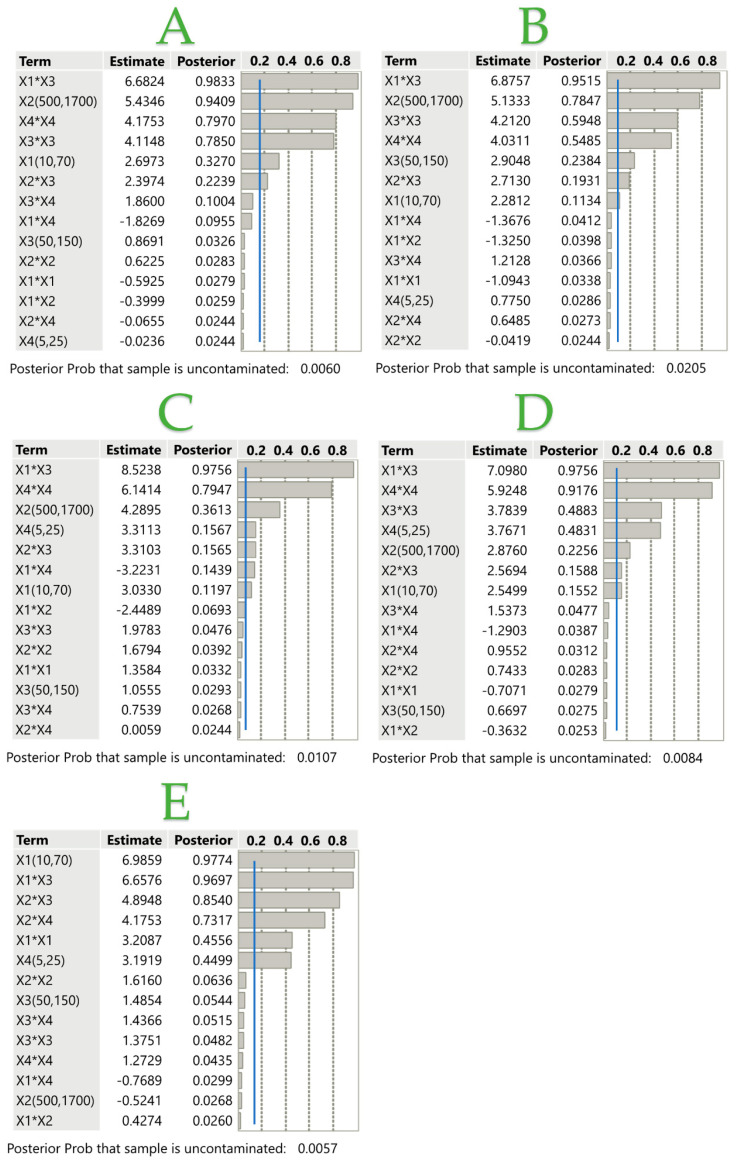
Bayes plots of transformed estimates for TPC (**A**), TFC (**B**), FRAP (**C**), DPPH (**D**), and AHPA (**E**) assays. The posterior blue line determines which effects are active in the model, which translates to a *p*-value of <0.05. The note beneath the plot in the Bayes plot report gives the posterior probability that the sample is uncontaminated.

The Bayes plot computes posterior probabilities for all model terms using a Bayesian approach. The Posterior Prob is the probability, based on the priors and the data, that there are no active effects whatsoever. The remaining estimates, known as active estimates, are considered to be derived from a contaminating distribution with increased variance. The probability is small for all variables, <0.05, indicating that it is likely that there are active effects. In Figure 2, the Bayes plots for TPC (A), TFC (B), FRAP (C), DPPH (D), and AHPA (E) assays are presented. Figure 2 shows that the main factors that positively affect TPC are the combination of the liquid-to-solid ratio with temperature and the high-pressure values, whose effect on the extraction efficiency tends to reach its maximum, with their posterior values close to 1. The extraction time and temperature also have a significant positive influence, with the posterior being close to 0.8, while the ratio values and the pressure-temperature combination have posterior values between 0.2 and 0.3, having minimal influence on extraction performance in terms of TPC. The TFC is highly dependent on the combination of ratio and extraction temperature, followed by the pressure at higher and lower values. The pressure and extraction duration also positively affect the TFC recovery, along with the combination of pressure and temperature. The combination of ratio and temperature seems to be positively affecting FRAP, DPPH, and AHPA assays significantly, as their posterior probabilities are ~0.97 in each case. It should be noted that the combination of ratio and extraction duration is the only term that negatively affects the FRAP assay, while it has no considerable impact on any of the other assays.

### 2.3. Optimal Extraction Conditions

To achieve optimization, the desirability function was utilized to identify the maximum anticipated levels of TPC, TFC, and antioxidant activity, measured through FRAP, DPPH, and AHPA. The maximum values of the responses should be obtained when a relatively high pressure of 1500–1650 psi is applied, at a temperature of 150 °C, at a high liquid-to-solid ratio, and for an extended extraction time. With these parameters, a TPC of 25.83 mg GAE/g dw and a TFC of 20.13 mg RtE/g dw are anticipated, while the antioxidant capacity of the extract should be 131.13, 143.21, and 253.24 μmol AAE/g dw, as determined by FRAP, DPPH, and AHPA assays, respectively. Additional information about the optimal extraction parameters is provided in Table S2.

### 2.4. Multiple Factor Analysis (MFA) and Multivariate Correlation Analysis (MCA)

**Figure 3:**
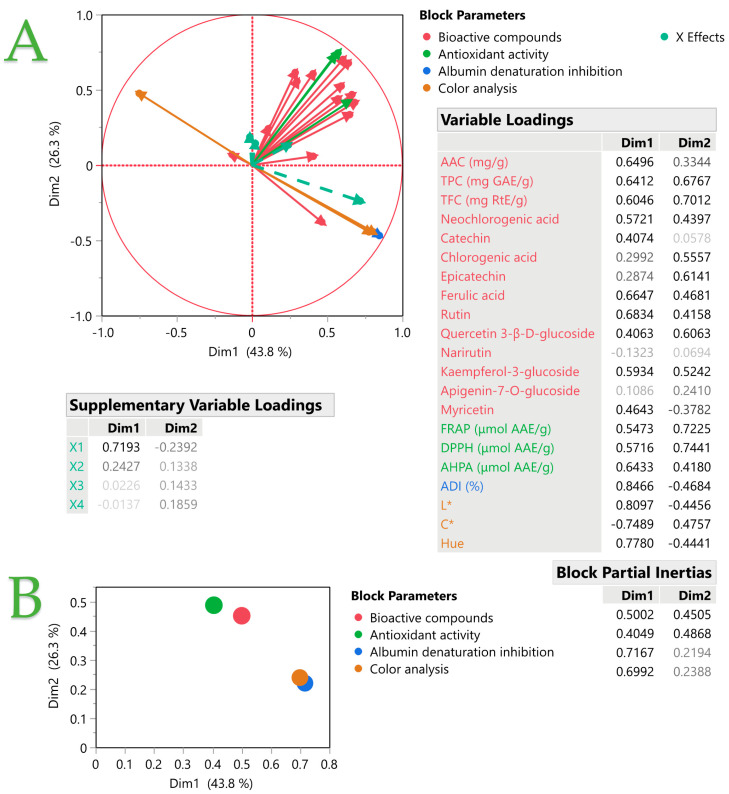
Multiple factor analysis for the measured variables. Plot (**A**) displays the factor scores of each variable, and plot (**B**) shows the block partial inertia. Inset tables include variable loadings and colors represent the block parameters of the variables.

Multiple Factor Analysis (MFA) is a technique that extends Principal Components Analysis to data sets with multiple variables measured on the same items. It allows the comparison of the views of different participants by transforming the variables into orthogonal factors. These factors reveal the similarities and differences among the items based on the participants ratings. We performed MFA to examine the relationships among the measured variables. Figure 3 shows the results of the MFA. Figure 3A shows the factor scores of each measurement variable on the first two dimensions, which explain 43.8% and 26.3% of the total variance, respectively. The plot also includes examined variables (*X*_1_–*X*_4_), which are marked according to their levels. The plot reveals blocks of items that are similar (or familiar) to each other based on their proximity in the factor space. Figure 3B Plot (B) shows the block partial inertia, which measures the contribution of each set of variables to each dimension. It is calculated by multiplying the block partial inertia by the eigenvalue and dividing by 100. This helps to assess how much each block contributes to the overall structure of the data. The inset tables provide the variable loadings, which indicate the correlations between the variables and the components. The colors in both plots represent the block parameters of the variables, which reflect their relative importance in each set of variables.

Bioactive compounds play a crucial role in our health, and some of them exhibit anti-inflammatory properties. Phenolic acids, such as neochlorogenic acid and chlorogenic acid, have been reported to exhibit strong medicinal activities, including antioxidant, anti-inflammatory, antifungal, and antibacterial properties, among others [2]. Catechins, which are typically found in tea leaves, belong to the flavonoid family and exhibit potent antioxidant properties and notable physiological activity [36]. They are also recognized for their significant anti-inflammatory, antioxidant, and chemopreventive properties [37]. Myricetin is also classified as a flavonol, which is a type of flavonoid [2]. Myricetin is currently recognized for its various biological properties, including antioxidant [37] and anti-inflammatory [37] activity. Another compound with promising bioactive effects is quercetin and its derivatives, which are naturally occurring flavonols [2]. Quercetins have been reported to possess antioxidant and anti-inflammatory activity [2]. All these compounds are present in MO leaf extracts emerging via PLE, as already presented in Table 3.

**Figure 4:**
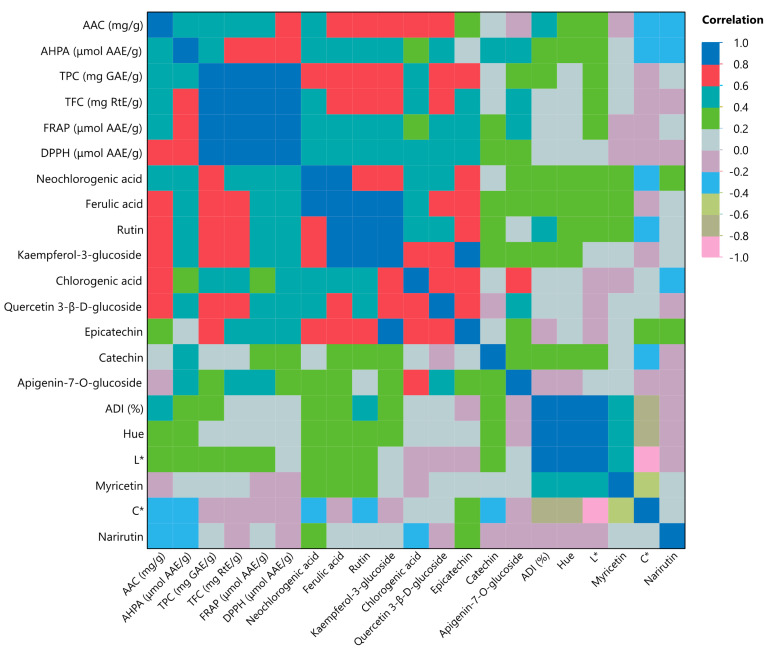
Multivariate correlation analysis of measured variables.

Considering Figure 3 and the MFA, it is noticed that when the liquid-to-solid ratio increases, which means the amount of solid MO used during extraction is reduced, this leads to an increase in the ADI. Hence, it could mean that the high solid amount in the extraction leads to high solid dispersion into the solution, which leads to higher stability of the solid phase, thus proving to exhibit pre-inflammatory activity [38]. On the other hand, when the solid amount is decreased, the intensity of the extract color also decreases. The MO extracts through PLE exhibit considerable albumin denaturation inhibition, and this could be attributed to the presence of flavonoids [39], and, more specifically, myricetin and catechin in the extracts. Another study by Shervington et al. [40] indicated that the high anti-inflammatory activity in MO leaves was due to its flavonoids, and the main flavonoids they determined were myricetin and quercetin, along with kaempferol. Moreover, the antioxidant activity of the extract displays a positive correlation with all the bioactive compounds found in it. These observations are further reinforced by the multivariate correlation analysis (MCA) analysis in Figure 4. In Figure S12, a multivariate color map of the *p*-values of measured variables is illustrated, where the pink color denotes the statistically significant differences (*p* < 0.05) of the variables. As can be seen, TPC, TFC, FRAP, and DPPH are all positively correlated with each other, while they all correlate negatively with coordinate C*. This leads to the conclusion that when the color of the extracts was intense, the measured TCP, TFC, FRAP, and DPPH values were low. It is also noteworthy that the presence of myricetin, AAC, and AHPA negatively correlates with coordinate C* in color determination. Enhancing this observation, coordinate C* also correlates negatively with neochlorogenic acid, which is proven to be the main compound in MO extracts determined via HPLC-DAD.

### 2.5. Partial Least Squares (PLS) Analysis

**Figure 5:**
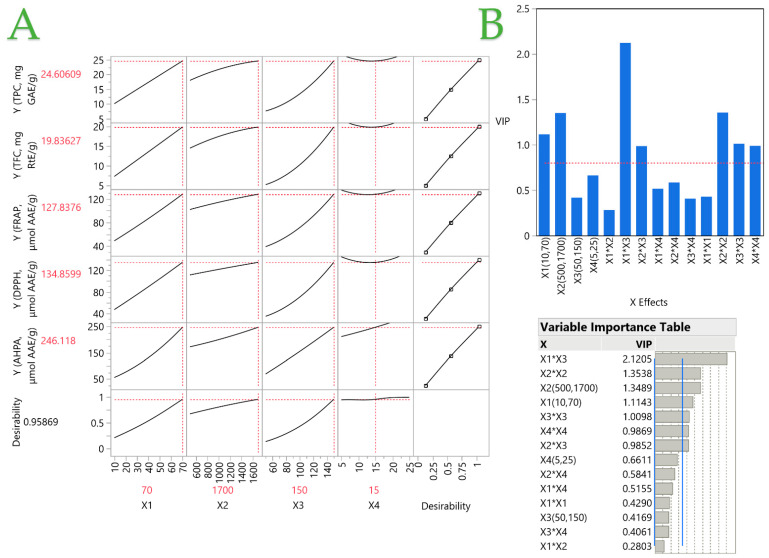
The PLS prediction profiler of each variable and the desirability function for optimizing *M. oleifera* extracts are shown in plot (**A**), while the Variable Importance Plot (VIP) option graph with the VIP values for each predictor variable is shown in plot (**B**). The VIP scores are also displayed in the VIT. A red dashed line in the plot (or a blue line in the VIT) at 0.8 indicates the significance level of each variable.

To determine the significance of the extraction parameters (*X*_1_, *X*_2_, *X*_3_, and *X*_4_), a PLS model was applied. The utilization of the PLS model to generate a correlation loading plot, as illustrated in Figure 5, visually represents the influence of extraction conditions on MO leaves. When the projection factor is greater than 0.8, it denotes a more significant contribution from the specified variable. In this case, it can be concluded that the optimal liquid-to-solid ratio (*X*_1_) is the highest one, namely 70, in all cases, while the optimal pressure value (*X*_2_) is 1700 psi, the temperature (*X*_3_) that leads to the maximum yield is 150 °C, and the optimal duration of the extraction (*X*_4_) is 15 min.

The experimental results and the PLS model predictions are in excellent agreement, as evidenced by the high correlation coefficient of 0.9985 and the high determination coefficient (R^2^) of 0.997. A *p*-value of <0.0001 indicates that the deviations between the observed and predicted values are not significant. Table 4 displays the predicted values of PLS together with the corresponding experimental values for TPC, TFC, and antioxidant assays, employing the optimal conditions. Table 5 summarizes the values of several individual antioxidant compounds and the color properties of the extract under optimal extraction conditions.

The optimal TPC was measured to be 24.28 mg GAE/g dw, which is in strong agreement with the PLS model value. Rodríguez-Pérez et al. [17] also reported a TPC of 24.3 mg GAE/g dry leaf on MO maceration extracts, while the TPC obtained by ultrasound-assisted extraction was ~26% lower. Naeem et al. [41] determined 12.28–13.65 mg GAE/g dw on MO leaves through solvent extraction, almost ~78% lower than the yield given via PLE. Pollini et al. [42] employed ultrasound-assisted extraction on MO leaves, and the TPC they measured was also ~82% lower than in our case. Nobossé et al. [43] also determined 21.6 mg GAE/g dw in 60-day leaf aqueous extracts through stirring, thus enhancing the notion that PLE is an effective process to extract polyphenols from MO leaves. The TFC was determined at 17.20 mg RtE/g dw, which is close to the PLS model, too. The FRAP value of the optimal extract was 122.97 μmol AAE/g dw, once again close to the PLS model. Karageorgou et al. [44] also reported a similar FRAP value of 131.67 μmol AAE/g dw of MO leaf extract. Karthivashan et al. [45] reported a value that was ~17% lower than ours. The DPPH value was measured at 127.21 μmol AAE/g dw, which is also not far from the predicted PLS value. The AHPA was determined to be 230.14 μmol AAE/g dw, which was also close to the predicted one.

Sreelatha and Padma [16] reported a similar AAC value of 5.81 and 6.60 mg/g dw on MO tender and mature leaves, respectively. The color intensity in the optimal extract was weak, as the highest ratio was applied. Therefore, the extract exhibits high albumin denaturation inhibition, confirming the statistical models applied and discussed above. The most abundant polyphenol determined in the optimal extract was neochlorogenic acid, with a quantity of 5.59 mg/g dw, followed by kaempferol-3-glucoside and myricetin, at 3.32 and 2.27 mg/g dw, respectively. Sibhat et al. [46] determined less than 2 mg/g of both neochlorogenic acid and kaempferol-3-glucoside in *Moringa stenopetala* leaves.

## 3. Materials and Methods

### 3.1. Chemicals and Reagents

Phosphate buffer solution, 2,2-diphenyl-1-picrylhydrazyl (DPPH), hydrochloric acid, *L*-ascorbic acid, albumin, ascorbic acid, methanol, aluminum chloride, 2,4,6-tris(2-pyridyl)-*s*-triazine (TPTZ), trichloroacetic acid, and all standards for HPLC polyphenol identification and quantification were purchased from Sigma-Aldrich (Darmstadt, Germany). Gallic acid, Folin–Ciocalteu reagent, and ethanol were from Panreac Co. (Barcelona, Spain). Labkem (Barcelona, Spain) provided the acetonitrile. Iron (III) chloride was obtained from Merck (Darmstadt, Germany). Formic acid (98%) and sodium carbonate (anhydrous) were bought from Penta (Prague, Czech Republic). Hydrogen peroxide (35% *v*/*v*) was from Chemco (Malsch, Germany). Deionized water was used for all conducted experiments.

### 3.2. Instrumentation

A Biobase BK-FD10 (Jinan, China) freeze-dryer was used to lyophilize MO leaves. A Pressurized Liquid Extraction (PLE) system (Fluid Management Systems, Inc., Watertown, MA, USA) was used to conduct all extractions. A Shimadzu UV-1900i Double-beam UV–Vis Spectrophotometer (Kyoto, Japan) was used for all spectrophotometric analyses. A Shimadzu CBM-20A liquid chromatograph and a Shimadzu SPD-M20A diode array detector (DAD) (Shimadzu Europa GmbH, Duisburg, Germany) were used for the quantification of individual polyphenols. The compounds were separated into a Phenomenex Luna C18(2) column from Phenomenex Inc. in Torrance, CA, USA, kept at 40 °C (100 Å, 5 μm, 4.6 mm × 250 mm). A colorimeter (Lovibond CAM-System 500, The Tintometer Ltd., Amesbury, UK) was used to determine CIELAB parameters (L*, a*, and b*) from the aqueous MO extracts.

### 3.3. Collection and Handling of MO Leaves

The cultivation of MO seedlings took place in the Krya Vrisi area of the Karditsa region, located in central Greece. The coordinates of the area are 39°19′6.97″ N and 21°52′39.16″ E, with an elevation of 131 m, as reported by Google Earth version 7.0.1.8244-beta, Google, Inc., Cambridge, MA, USA. This region possesses a moderate climate characterized by a hot and arid summer period, whereby the average yearly temperature stands at 15.7 °C, while the mean monthly temperatures for August and September are recorded at 29.1 °C and 23.5 °C, respectively. The soils in this area consist of sandy loam, which has a slightly acidic pH of 6.4. They have a low content of organic matter and have limited availability of macronutrients and boron. However, they have a normal availability of micronutrients. Fertilization was not administered; rather, drip irrigation was implemented on a biweekly basis, resulting in a total irrigation volume of 3800 m^3^·ha^–1^ throughout the entire season. Leaves that were two months old were gathered in the morning on September 2023. The leaves were transported to the laboratory 30 min after being harvested. There, they were meticulously rinsed with tap water and subjected to freeze-drying for 24 h. Subsequently, they were ground into a fine powder with an average diameter of 400–800 μm, which was kept at –40 °C until further analysis.

### 3.4. MO Leaves Extraction Procedure

The extraction procedure involving PLE was based on a previous study [47]. To identify the optimal conditions for the recovery of bioactive compounds from MO leaves, different combinations of extraction conditions were used, as shown in Table 6. In all cases, MO leaves were mixed with 40 mL of solvent (water).

### 3.5. Optimization with Response Surface Methodology (RSM) and Experimental Design

An RSM approach was implemented to maximize bioactive compound extraction and examine the antioxidant properties of MO leaf extracts. To assess the antioxidant and biological activities of MO leaves and to recover bioactive compounds in the most efficient way, we used the RSM technique. Thus, the principal objective of the design was to maximize the levels of these values in an effective manner. This was accomplished by optimizing the liquid-to-solid ratio (*R*, mL/g), along with PLE conditions, including pressure (*P*, psi), temperature (*T*, °C), and extraction duration (*t*, min). The extraction optimization process was determined based on a Box–Behnken design with a main impact screening arrangement, Considering optimization, a total of 27 design point experiments, leading to a screening main effect, were conducted. In accordance with the experimental design, the process variables had to be established at five distinct levels. The overall significance of the model (R^2^, *p*-value) and the significance of the model coefficients (equations) were determined using analysis of variance (ANOVA) and summary-of-fit tests, with a minimum level of 95%. Additionally, the response variable was predicted as a function of the examined independent factors using a second-order polynomial model, as illustrated in Equation (1):

(1) $$Y_{k}=\beta_{0}+\sum_{i=1}^{2}\beta_{i}X_{i}+\sum_{i=1}^{2}\beta_{\mathrm{ii}}X_{i}^{2}+\sum_{i=1}^{2}\sum_{j=i+1}^{3}\beta_{\mathrm{ij}}X_{i}X_{j}$$

where *X_i_* and *X_j_* represent the independent variables and *Y_k_* defines the predicted response variable. The model linear, quadratic, and interaction terms are represented by the intercept and regression coefficients, *β*_0_, *β_i_*, *β_ii_*, and *β_ij_*, respectively.

By identifying the largest peak area and assessing the effect of a significant independent variable on the response, the RSM was utilized. In order to represent the model equation graphically, three-dimensional surface response graphs were generated.

### 3.6. Bioactive Compounds Determination

#### 3.6.1. Total Polyphenol Content (TPC)

The total polyphenol content (TPC) of the extracts was calculated according to a former study [27]. In brief, 0.4 mL of sample were mixed with 0.4 mL of Folin–Ciocalteu reagent and after 2 min, 3.2 mL of 5% *w*/*v* aqueous sodium carbonate solution was added in a 5-mL Eppendorf tube. The absorbance was recorded at 740 nm after incubation at 40 °C for 20 min. The total polyphenol concentration (*C*_TP_) was calculated from a gallic acid calibration curve. TPC was determined as mg gallic acid equivalents (GAE) per g of dry weight (dw), according to Equation (2):

(2) $$\mathrm{TPC}\;(\mathrm{mg}\;\mathrm{GAE}/g\;\mathrm{dw})=\frac{C_{\mathrm{TP}}\;\times V}{w}$$

where *w* is the dry weight of the sample (in g) and V is the extraction medium volume (in L).

#### 3.6.2. Total Flavonoid Content (TFC)

The total flavonoid content (TFC) was determined based on a previously established technique [48]. In brief, a volume of 0.1 mL of properly diluted sample was mixed with 0.86 mL of aqueous ethanol (35% *v*/*v*) and 0.04 mL of a reagent that included 5% (*w*/*v*) aluminum chloride and 0.5 M sodium acetate. The mixture was kept at ambient temperature for 30 min before measuring the absorbance at 415 nm. A rutin (quercetin 3-*O*-rutinoside) calibration curve (30–300 mg/L in methanol) was used to measure the total flavonoid concentration (*C*_TFn_). The TFC was expressed as mg rutin equivalents (RtE) per g dry weight (dw), as portrayed in Equation (3):

(3) $$\mathrm{TFC}\;(\mathrm{mg}\;\mathrm{RtE}/g\;\mathrm{dw})=\frac{C_{\mathrm{TFn}}\;\times \;V}{w}$$

where *V* denotes the extraction medium volume (in L) and *w* is the dry weight of the sample (in g).

#### 3.6.3. Individual Polyphenolic Compound Quantification

To quantify individual polyphenolic compounds, high-performance liquid chromatography (HPLC) was utilized, as established in our previous research [48]. A HPLC-DAD system was employed for the analysis of MO extracts, and the compounds were separated into column sat 40 °C. The mobile phase included 0.5% aqueous formic acid (A) and 0.5% formic acid in acetonitrile (B). The gradient program required: initially from 0 to 40% B, then to 50% B in 10 min, to 70% B in another 10 min, and then constant for 10 min. The mobile phase had a flow rate of 1 mL/min. The absorbance spectrum and retention time were compared to those of pure standards in order to achieve identification and then quantification through calibration curves (0–50 μg/mL).

#### 3.6.4. Ascorbic Acid Content (AAC)

Ascorbic acid content (AAC) was determined with a previously established method [48] and expressed as mg/g dw. Descriptively, 0.1 mL of the sample extract along with 0.5 mL of 10% (*v*/*v*) Folin–Ciocalteu reagent were mixed with 0.9 mL of 10% (*w*/*v*) trichloroacetic acid in an Eppendorf tube. After a 10 min wait at ambient temperature, the absorbance was measured at 760 nm.

### 3.7. Antioxidant Activity of the Extracts

#### 3.7.1. Ferric Reducing Antioxidant Power (FRAP) Assay

A previously described methodology by Shehata et al. [49] was employed to evaluate the FRAP values of the extracts. In a 2 mL Eppendorf tube, 0.1 mL of the properly diluted sample was mixed with 0.1 mL of FeCl_3_ solution (4 mM in 0.05 M HCl). The mixture was incubated at 37 °C for 30 min, with 1.8 mL of TPTZ solution (1 mM in 0.05 M HCl) being immediately added right after, and the absorbance was measured after 5 min at 620 nm. The ferric reducing power (*P*_R_) was measured using an ascorbic acid calibration curve (*C*_AA_) in 0.05 M HCl with ranging values (0.05–0.5 mM). The *P*_R_ was calculated as μmol of ascorbic acid equivalents (AAE) per g of dw using Equation (4):

(4) $$P_{R}\;(\mu \mathrm{mol}\;\mathrm{AAE}/g\;\mathrm{dw})=\frac{C_{AA}\;\times \;V}{w}$$

where *V* stands for the volume of the extraction medium (in L), and *w* stands for the dry weight of the sample (in g).

#### 3.7.2. DPPH^•^ Antiradical Activity Assay

The recovered bioactive compounds from the MO were assessed for their antiradical activity (*A*_AR_) using a DPPH^•^ method described by Shehata et al. [49]. In brief, 0.05 mL of a properly diluted sample was mixed with a quantity of 1.95 mL of a 100 μM DPPH^•^ solution in methanol. The solution was left in the dark at room temperature for 30 min. Following that, the absorbance was measured at 515 nm. A blank sample consisting of methanol and DPPH^•^ solution was used, and the absorbance was immediately measured at 515 nm. The inhibition percentage was calculated according to Equation (5):

(5) $$\mathrm{Inhibition}\;\left(\%\right)=\frac{A_{515\left(i\right)\;}-A_{515\left(f\right)}}{A_{515\left(i\right)}}\;\times \;100$$

Equation (6) involving an ascorbic acid calibration curve (*C*_AA_) was applied to assess the antiradical activity (*A*_AR_) in μmol AAE per g of dw:

(6) $$A_{\mathrm{AR}}\left(\mu \mathrm{mol}\;\mathrm{AAE}/g\;\mathrm{dw}\right)=\frac{C_{\mathrm{AA}}\;\times \;V}{w}$$

where *V* stands for the extraction medium volume (in L), and *w* stands for the dry weight of the sample (in g).

#### 3.7.3. Anti-Hydrogen Peroxide Activity (AHPA)

To identify the H_2_O_2_ inhibition activity of MO leaf extracts, our previous methodology [27] was used. A quantity of 0.6 mL of a H_2_O_2_ solution (40 mM, diluted in phosphate buffer with pH 7.4) was combined with 0.4 mL of the extract in an Eppendorf tube. Then, after 10 min at ambient temperature, the absorbance was measured at 230 nm. Equation (7) was used to assess the inhibition capacity of the H_2_O_2_:

(7) $$\mathrm{Inhibition}\;(\%)=\frac{A_{o}-({A-A}_{c})}{A_{o}}\;\times \;100$$

where *A*_o_ denotes the absorbances of the blank solution, *A*_c_ the extract solution in the absence of hydrogen peroxide, and *A* the sample.

An ascorbic acid calibration curve (*C*_AA_, 50–500 μmol/L in 0.05 M HCl) and the following Equation (8) were utilized to determine the AHPA as μmol AAE per g of dw:

(8) $$\mathrm{AHPA}\;(\mu \mathrm{mol}\;\mathrm{AAE}/g\;\mathrm{dw})=\frac{C_{AA}\;\times \;V}{w}$$

where *V* is the volume of the extraction medium (in L) and *w* is the dry weight of the sample.

### 3.8. Biological and Physicochemical Properties of MO Extracts

#### 3.8.1. In Vitro Albumin Denaturation Inhibition (ADI)

The albumin denaturation assay with slight modifications, as described elsewhere [50] was used to evaluate the in vitro albumin denaturation inhibition of MO extracts. Briefly, a mixture containing 1% *w*/*v* albumin in PBS (pH = 7.4) (mixture A), respectively, was prepared. Then, 0.6 mL of mixture A was mixed with 0.4 mL of 1:20 *v*/*v* diluted sample extract in a tube, and then the tubes were incubated for 20 min at 37 °C. Afterwards, the mixture was heated at 70 °C for 20 min. The absorbance was then recorded at 660 nm. A control sample was prepared, consisting of mixture A and 0.4 mL of water. Also, a blank solution was prepared by mixing 0.6 mL of water with 0.4 mL of each diluted sample extract. The inhibition of protein denaturation was assessed according to the following Equation (9):

(9) $$\mathrm{Inhibition}\;(\%)=\frac{{A_{\mathrm{control}}-\;(A}_{\mathrm{sample}}{-A}_{\mathrm{blank}})}{A_{\mathrm{control}}}\times \;100$$

#### 3.8.2. Color Determination

The CIELAB color determination of MO extracts was performed using a previously established methodology [27], where the CIELAB parameters (L*, a*, and b*) were measured. The lightness of a color, ranging from 0 (representing black) to 100 (representing white), is given by the L* value. The degree of redness (negative values) or greenness (positive values) in a color is specified by the a* value. Similarly, the extent of yellowness (negative values) or blueness (positive values) in a color is measured by the b* value. The measure of color intensity is denoted by *C_ab_* or C* (chroma, saturation). The psychological coordinate Chroma ($C_{ab}^{*}$) and the hue angle ($h_{ab}^{o}$ or *H*) were determined by the following Equations (10) and (11):

(10) $$C_{ab}^{*}=\sqrt{{\left(a^{*}\right)}^{2}+{\left(b^{*}\right)}^{2}}$$

(11) $$h_{ab}^{o}=\arctan\left(\frac{b^{*}}{a^{*}}\right)$$

### 3.9. Statistical Analysis

The statistical analysis pertaining to response surface methodology and distribution analysis was conducted via JMP^®^ Pro 16 software (SAS, Cary, NC, USA). The quantitative analysis was conducted three times, and the extraction procedures were repeated at least twice for each batch of ΜO extracts. The normality of the data was evaluated using the Kolmogorov-Smirnov test. In order to determine if there were any significant differences, a one-way analysis of variance (ANOVA) was performed, utilizing a multiple comparison test known as Tukey HSD. The outcomes are presented in the format of averages and measures of variability. The statistical analyses of Bayes plot analysis (BPA), multiple factor analysis (MFA), multivariate correlation analysis (MCA), and partial least squares (PLS) analysis were performed using JMP^®^ Pro 16 software.

## 4. Conclusions

In conclusion, the study highlights the effectiveness of PLE in maximizing the extraction yield of bioactive compounds from MO leaves. By optimizing key PLE parameters such as liquid-to-solid ratio (70 mL/g), temperature (150 °C), pressure (1700 psi), and extraction duration (15 min), and employing a cost-effective and readily available solvent (deionized water), significant quantities of total polyphenols (24.61 mg GAE/g dw), total flavonoids (19.84 mg RtE/g dw), and ascorbic acid content (4.62 mg/g dw) were successfully obtained. The antioxidant activities evaluated through various methods underscore the potential health benefits of these extracts. Moreover, the observed albumin denaturation inhibition underscores the therapeutic potential of MO leaves. These findings support the use of PLE as a sustainable and efficient extraction method to unlock the valuable bioactive properties of MO leaves, with wide-ranging applications across industries including pharmaceuticals, nutraceuticals, and functional foods.

## Figures and Tables

**Table 1:** Experimental outcomes for the combinations of the four independent variables into consideration and the dependent variables responses.

Design Point	Independent Variables				Responses				
	*X*_1_ (*R*, mL/g)	*X*_2_ (*P*, psi)	*X*_3_ (*T*, °C)	*X*_4_ (*t*, min)	TPC ^1^	TFC ^2^	FRAP ^3^	DPPH ^4^	AHPA ^5^
1	0 (40)	0 (1100)	−1 (50)	−1 (5)	19.45	12.69	78.46	90.10	99.98
2	0 (40)	1 (1700)	0 (100)	1 (25)	15.44	12.21	91.06	91.89	127.34
3	0 (40)	0 (1100)	0 (100)	0 (15)	9.62	7.42	48.97	43.83	80.97
4	1 (70)	0 (1100)	0 (100)	1 (25)	13.52	9.85	83.38	81.36	186.35
5	0 (40)	−1 (500)	−1 (50)	0 (15)	11.73	8.85	70.77	75.34	169.08
6	−1 (10)	0 (1100)	0 (100)	1 (25)	15.09	11.73	108.13	97.25	132.40
7	1 (70)	−1 (500)	0 (100)	0 (15)	12.04	9.94	78.90	69.12	153.26
8	0 (40)	0 (1100)	−1 (50)	1 (25)	15.58	11.95	77.86	102.22	95.13
9	1 (70)	1 (1700)	0 (100)	0 (15)	17.24	12.01	84.66	84.38	180.71
10	−1 (10)	0 (1100)	−1 (50)	0 (15)	19.91	14.41	91.33	91.19	110.70
11	−1 (10)	0 (1100)	1 (150)	0 (15)	8.82	6.16	42.98	33.55	43.11
12	0 (40)	1 (1700)	−1 (50)	0 (15)	17.47	12.56	83.76	81.48	86.45
13	0 (40)	0 (1100)	0 (100)	0 (15)	9.61	7.75	48.93	44.97	80.98
14	0 (40)	0 (1100)	1 (150)	−1 (5)	16.68	15.62	79.81	75.28	97.82
15	0 (40)	0 (1100)	1 (150)	1 (25)	20.10	18.72	90.97	117.71	142.13
16	−1 (10)	1 (1700)	0 (100)	0 (15)	11.90	10.58	72.58	66.54	105.79
17	1 (70)	0 (1100)	−1 (50)	0 (15)	9.00	6.37	39.67	48.97	69.15
18	−1 (10)	−1 (500)	0 (100)	0 (15)	7.46	5.12	39.46	49.05	93.06
19	1 (70)	0 (1100)	1 (150)	0 (15)	24.10	19.89	124.29	131.28	229.38
20	0 (40)	−1 (500)	0 (100)	−1 (5)	12.08	9.29	63.53	71.69	131.97
21	0 (40)	1 (1700)	0 (100)	−1 (5)	13.81	9.78	70.24	65.98	62.90
22	0 (40)	1 (1700)	1 (150)	0 (15)	22.28	16.48	91.48	105.72	130.82
23	0 (40)	−1 (500)	0 (100)	1 (25)	12.56	8.94	79.56	81.16	72.48
24	−1 (10)	0 (1100)	0 (100)	−1 (5)	12.42	9.66	61.96	65.17	46.86
25	1 (70)	0 (1100)	0 (100)	−1 (5)	18.01	12.11	87.49	74.72	127.12
26	0 (40)	0 (1100)	0 (100)	0 (15)	9.63	7.46	48.69	45.40	81.01
27	0 (40)	−1 (500)	1 (150)	0 (15)	7.06	5.89	40.84	48.63	75.27

^1^ Total polyphenol content (TPC) in mg GAE/g dw. ^2^ Total flavonoid content (TFC) in mg RtE/g dw. ^3^ ferric reducing antioxidant power (FRAP) in μmol AAE/g dw. ^4^ 2,2-diphenyl-1-picrylhydrazyl (DPPH) in μmol AAE/g dw. ^5^ Anti-hydrogen peroxide activity (AHPA) in μmol AAE/g dw.

**Table 2:** The coded and actual values of the four independent variables under inquiry, as well as the experimental concentrations of ascorbic acid, albumin denaturation inhibition, and color analysis coordinates.

Design Point	Independent Variables				Responses							
	*X*_1_ (*R*, mL/g)	*X*_2_ (*P*, psi)	*X*_3_ (*T*, °C)	*X*_4_ (*t*, min)	AAC ^1^	ADI ^2^	L*	a*	b*	C*	Hue	Color ^3^
1	0 (40)	0 (1100)	−1 (50)	−1 (5)	3.80	42.30	71.9	−2.2	23.6	23.7	95.4	
2	0 (40)	1 (1700)	0 (100)	1 (25)	2.60	38.35	80.3	−0.9	12.4	12.5	94.3	
3	0 (40)	0 (1100)	0 (100)	0 (15)	1.83	39.88	74.9	−1.5	15.3	15.4	95.4	
4	1 (70)	0 (1100)	0 (100)	1 (25)	2.80	39.95	81.1	−0.7	7.5	7.5	95.1	
5	0 (40)	−1 (500)	−1 (50)	0 (15)	3.36	35.56	68.5	−0.9	29.1	29.2	91.9	
6	−1 (10)	0 (1100)	0 (100)	1 (25)	1.33	20.93	69.7	1.5	39.6	39.6	87.9	
7	1 (70)	−1 (500)	0 (100)	0 (15)	3.26	39.11	71.0	−2.7	20.5	20.7	97.5	
8	0 (40)	0 (1100)	−1 (50)	1 (25)	3.25	20.46	59.2	8.5	29.3	30.5	73.9	
9	1 (70)	1 (1700)	0 (100)	0 (15)	3.30	38.39	78.5	−1.5	7.7	7.9	100.8	
10	−1 (10)	0 (1100)	−1 (50)	0 (15)	2.35	27.83	70.2	−0.9	37.0	37.0	91.4	
11	−1 (10)	0 (1100)	1 (150)	0 (15)	1.57	19.95	64.3	7.2	36.5	37.2	78.8	
12	0 (40)	1 (1700)	−1 (50)	0 (15)	2.47	37.88	77.6	−2.2	12.7	12.9	100.0	
13	0 (40)	0 (1100)	0 (100)	0 (15)	1.73	40.70	77.4	−2.2	14.2	14.4	98.9	
14	0 (40)	0 (1100)	1 (150)	−1 (5)	3.16	39.93	72.8	−3.0	25.2	25.4	96.8	
15	0 (40)	0 (1100)	1 (150)	1 (25)	3.92	37.35	71.5	−2.9	23.4	23.5	97.1	
16	−1 (10)	1 (1700)	0 (100)	0 (15)	1.46	28.45	65.4	4.8	42.0	42.2	83.4	
17	1 (70)	0 (1100)	−1 (50)	0 (15)	2.98	40.18	79.3	−2.0	8.0	8.2	104.1	
18	−1 (10)	−1 (500)	0 (100)	0 (15)	1.25	19.98	60.1	7.2	38.5	39.2	79.4	
19	1 (70)	0 (1100)	1 (150)	0 (15)	4.77	37.88	75.7	0.7	12.2	12.2	86.9	
20	0 (40)	−1 (500)	0 (100)	−1 (5)	2.95	40.61	75.4	−0.9	20.3	20.3	92.6	
21	0 (40)	1 (1700)	0 (100)	−1 (5)	2.38	41.18	76.2	−0.4	14.0	14.0	91.6	
22	0 (40)	1 (1700)	1 (150)	0 (15)	3.83	35.53	72.1	−0.4	24.2	24.2	90.9	
23	0 (40)	−1 (500)	0 (100)	1 (25)	3.03	35.34	69.9	0.4	29.4	29.4	89.2	
24	−1 (10)	0 (1100)	0 (100)	−1 (5)	1.76	21.44	58.8	13.8	34.9	37.5	68.4	
25	1 (70)	0 (1100)	0 (100)	−1 (5)	3.04	33.32	76.1	0.4	14.4	14.4	88.4	
26	0 (40)	0 (1100)	0 (100)	0 (15)	1.77	37.31	75.2	−0.4	14.0	14.0	91.6	
27	0 (40)	−1 (500)	1 (150)	0 (15)	3.87	30.85	67.6	2.2	30.6	30.7	85.8	

^1^ Ascorbic acid content (AAC) in mg/g dw. ^2^ Albumin denaturation inhibition (ADI) in %. ^3^ Table cells were filled with the corresponding color of the extract using the proper HEX code, corresponding to the L*, a*, and b* values measured.

**Table 3:** Coded and actual values of the four independent variables were evaluated along with the experimental polyphenol concentration, in mg/g dw.

Design Point	Independent Variables				Responses										
	*X*_1_ (*R*, mL/g)	*X*_2_ (*P*, psi)	*X*_3_ (*T*, °C)	*X*_4_ (*t*, min)	NCA	CA	CGA	EC	FA	RT	Q3G	NRT	K3G	A7G	MYC
1	0 (40)	0 (1100)	−1 (50)	−1 (5)	6.13	0.95	1.62	0.40	0.37	0.94	3.04	1.02	2.83	0.08	1.16
2	0 (40)	1 (1700)	0 (100)	1 (25)	4.42	0.42	0.17	0.17	0.08	0.16	0.98	0.35	0.67	n.d. *	0.32
3	0 (40)	0 (1100)	0 (100)	0 (15)	2.50	0.42	0.11	0.09	0.06	0.12	0.77	0.28	0.38	n.d.	1.96
4	1 (70)	0 (1100)	0 (100)	1 (25)	2.95	3.40	0.23	0.11	0.12	0.28	0.54	0.25	0.81	n.d.	0.47
5	0 (40)	−1 (500)	−1 (50)	0 (15)	2.03	0.27	0.78	0.15	0.10	0.21	1.54	0.02	0.93	0.09	0.25
6	−1 (10)	0 (1100)	0 (100)	1 (25)	3.51	0.42	0.38	0.14	0.16	0.36	0.56	0.20	0.98	n.d.	0.55
7	1 (70)	−1 (500)	0 (100)	0 (15)	3.78	1.82	0.56	0.17	0.33	0.73	0.99	0.01	1.95	n.d.	0.91
8	0 (40)	0 (1100)	−1 (50)	1 (25)	3.28	0.37	1.37	0.17	0.12	0.22	1.57	0.02	0.98	n.d.	0.25
9	1 (70)	1 (1700)	0 (100)	0 (15)	6.81	1.03	1.06	0.31	0.35	0.78	2.86	0.35	2.61	n.d.	1.11
10	−1 (10)	0 (1100)	−1 (50)	0 (15)	5.77	1.42	0.46	0.39	0.30	0.65	1.82	1.01	1.90	n.d.	1.14
11	−1 (10)	0 (1100)	1 (150)	0 (15)	2.90	0.36	0.94	0.16	0.08	0.12	1.73	0.08	0.78	0.10	0.14
12	0 (40)	1 (1700)	−1 (50)	0 (15)	7.00	0.82	0.41	0.22	0.25	0.59	0.68	0.54	1.47	n.d.	0.98
13	0 (40)	0 (1100)	0 (100)	0 (15)	2.18	0.43	0.11	0.09	0.06	0.10	0.75	0.30	0.35	n.d.	1.89
14	0 (40)	0 (1100)	1 (150)	−1 (5)	4.76	0.63	1.00	0.23	0.22	0.51	2.38	0.18	1.86	0.06	0.69
15	0 (40)	0 (1100)	1 (150)	1 (25)	7.07	0.94	1.81	0.34	0.29	0.60	3.67	0.18	2.49	0.09	0.78
16	−1 (10)	1 (1700)	0 (100)	0 (15)	3.21	0.79	0.10	0.25	0.12	0.21	2.11	0.71	1.09	n.d.	0.47
17	1 (70)	0 (1100)	−1 (50)	0 (15)	4.63	0.50	0.30	0.14	0.17	0.41	0.34	0.31	0.98	n.d.	0.86
18	−1 (10)	−1 (500)	0 (100)	0 (15)	4.05	0.50	0.17	0.18	0.07	0.11	0.62	0.52	0.71	n.d.	0.27
19	1 (70)	0 (1100)	1 (150)	0 (15)	7.81	0.61	0.85	0.20	0.37	0.93	3.79	0.08	1.98	n.d.	1.29
20	0 (40)	−1 (500)	0 (100)	−1 (5)	6.26	0.71	0.39	0.07	0.20	0.46	1.12	0.56	1.35	n.d.	0.80
21	0 (40)	1 (1700)	0 (100)	−1 (5)	4.64	0.50	0.37	0.16	0.21	0.49	0.52	0.36	1.23	n.d.	0.80
22	0 (40)	1 (1700)	1 (150)	0 (15)	5.33	0.69	1.91	0.29	0.28	0.60	3.48	0.01	2.40	0.14	0.71
23	0 (40)	−1 (500)	0 (100)	1 (25)	3.47	0.43	0.39	0.15	0.17	0.40	0.72	0.27	1.13	n.d.	0.63
24	−1 (10)	0 (1100)	0 (100)	−1 (5)	4.61	0.26	0.17	0.20	0.11	0.22	0.87	1.19	0.77	n.d.	0.43
25	1 (70)	0 (1100)	0 (100)	−1 (5)	3.92	0.42	0.21	0.13	0.12	0.18	1.87	1.16	0.73	n.d.	0.50
26	0 (40)	0 (1100)	0 (100)	0 (15)	2.70	0.31	0.13	0.10	0.07	0.13	1.34	0.30	0.53	n.d.	1.26
27	0 (40)	−1 (500)	1 (150)	0 (15)	1.44	0.18	0.61	0.09	0.09	0.19	1.77	0.12	0.62	0.04	0.22

* n.d.: not detected. NCA: neochlorogenic acid; CA: catechin; CGA: chlorogenic acid; EC: epicatechin; FA: ferulic acid; RT: rutin; Q3G: quercetin 3-β-*D*-glucoside; NRT: narirutin; K3G: kaempferol-3-glucoside; A7G: apigenin-7-*O*-glucoside; MYC: myricetin.

**Table 4:** Maximum desirability for all variables using the partial least squares (PLS) prediction profiler under the optimal extraction conditions (*X*_1_: 1, *X*_2_: 1, *X*_3_: 1, and *X*_4_: 0).

Variables	PLS Model Values	Experimental Values
TPC (mg GAE/g dw)	24.61	24.28 ± 0.68
TFC (mg RtE/g dw)	19.84	17.20 ± 0.18
FRAP (μmol AAE/g dw)	127.84	122.97 ± 5.12
DPPH (μmol AAE/g dw)	134.86	127.21 ± 3.63
AHPA (μmol AAE/g dw)	246.12	230.14 ± 8.94

TPC: total polyphenol content; TFC: total flavonoid content; FRAP: ferric reducing antioxidant power; DPPH: 2,2-Diphenyl-1-picrylhydrazyl; AHPA: anti-hydrogen peroxide activity.

**Table 5:** Different parameters and polyphenols under optimal extraction conditions (*X*_1_: 1, *X*_2_: 1, *X*_3_: 1, and *X*_4_: 0).

Parameters	Optimal Extract		
AAC (mg/g dw)	4.62 ± 0.12		
ADI (%)	37.54 ± 0.36		
L*	78.1 ± 0.5		
a*	−2.2 ± 0.4		
b*	10.6 ± 0.8		
C*	10.8 ± 0.9		
Hue	101.8 ± 1.3		
Color			
**Polyphenolic Compounds (mg/g dw)**			
Neochlorogenic acid	5.59 ± 0.37		
Catechin	0.43 ± 0.03		
Chlorogenic acid	1.77 ± 0.08		
Epicatechin	0.23 ± 0.01		
Ferulic acid	0.67 ± 0.04		
Rutin	1.8 ± 0.07		
Quercetin 3-β-*D*-glucoside	0.59 ± 0.04		
Narirutin	0.48 ± 0.02		
Kaempferol-3-glucoside	3.32 ± 0.16		
Apigenin-7-*O*-glucoside	0.02 ± 0		
Myricetin	2.27 ± 0.13		
Total Identified	17.17 ± 0.95		

AAC: ascorbic acid content; ADI: albumin denaturation inhibition. Table cells were filled with the corresponding color of the extract using the proper HEX code, corresponding to the L*, a*, and b* values measured.

**Table 6:** The actual and coded levels of the independent variables were used to optimize the process.

Independent Variables	Code Units	Coded Variable Level		
		−1	0	1
Liquid-to-solid ratio (*R*, mL/g)	*X*_1_	10	40	70
Pressure (*P*, psi)	*X*_2_	500	1100	1700
Temperature (*T*, °C)	*X*_3_	50	100	150
Extraction time (*t*, min)	*X*_4_	5	15	25

## Data Availability

All related data and methods are presented in this paper. Additional inquiries should be addressed to the corresponding author.

## Supplementary Materials

The following supporting information can be downloaded at https://www.mdpi.com/article/10.3390/ijms25094628/s1.
