# Supplementary material for: Optimization of Pressurized Liquid Extraction (PLE) Parameters for Extraction of Bioactive Compounds from Moringa oleifera Leaves and Bioactivity Assessment
Source: Int J Mol Sci. 2024 Apr 24;25(9):4628. doi: 10.3390/ijms25094628 (PMC11083225; doi:10.3390/ijms25094628)
Supplement: Supplementary file 1 [file ijms-25-04628-s001.zip › ijms-2951033-supplementary.pdf]

Supplementary Material

Optimization of Pressurized Liquid Extraction (PLE) Parameters for Extraction of Bioactive Compounds from *Moringa oleifera* Leaves and Bioactivity Assessment

Theodoros Chatzimitakos, Vassilis Athanasiadis *, Konstantina Kotsou, Martha Mantiniotou, Dimitrios Kalompatsios, Ioannis Makrygiannis, Eleni Bozinou and Stavros I. Lalas

Department of Food Science and Nutrition, University of Thessaly, Terma N. Temponera Street,43100 Karditsa, Greece; tchatzimitakos@uth.gr (T.C.); kkotsou@agr.uth.gr (K.K.); mmantiniotou@uth.gr (M.M.); dkalompatsios@uth.gr (D.K.); ioanmakr1@uth.gr (I.M.); empozinou@uth.gr (E.B.); slalas@uth.gr (S.I.L.)

* Correspondence: vaathanasiadis@uth.gr; Tel.: +30-24410-64783

Table S1. Response surface methodology (RSM) employed mathematical models for optimizing the extraction of *M. oleifera*. These models exclusively included significant terms.

Responses	Second-Order Polynomial Equations (Models)	R^2 Predicted	R^2 Adjusted	*p*-Value	Eq.
TPC	$Y = 68.91 - 0.42X_1 - 0.03X_2 - 0.67X_3 - 1.18X_4 + 0.002X_1^2 + 0.0001X_2^2 + 0.002X_3^2 + 0.04X_4^2 - 0.0001X_1X_2 + 0.004X_1X_3 - 0.006X_1X_4 + 0.0001X_2X_3 - 0.0001X_2X_4 + 0.004X_3X_4$	0.9155	0.8169	0.0002	(S1)
TFC	$Y = 50.89 - 0.29X_1 - 0.02X_2 - 0.52X_3 - 0.94X_4 + 0.001X_1^2 + 0.0001X_2^2 + 0.001X_3^2 + 0.03X_4^2 - 0.0001X_1X_2 + 0.004X_1X_3 - 0.004X_1X_4 + 0.0001X_2X_3 + 0.0001X_2X_4 + 0.002X_3X_4$	0.9189	0.8242	0.0002	(S2)
FRAP	$Y = 295.64 - 1.93X_1 - 0.16X_2 - 2.5X_3 - 4.39X_4 + 0.02X_1^2 + 0.0001X_2^2 + 0.005X_3^2 + 0.21X_4^2 - 0.0005X_1X_2 + 0.02X_1X_3 - 0.04X_1X_4 + 0.0004X_2X_3 + 0.0001X_2X_4 + 0.006X_3X_4$	0.9396	0.8690	< 0.0001	(S3)
DPPH	$Y = 408.04 - 2.55X_1 - 0.2X_2 - 3.52X_3 - 8.13X_4 + 0.01X_1^2 + 0.0001X_2^2 + 0.009X_3^2 + 0.25X_4^2 - 0.0001X_1X_2 + 0.02X_1X_3 - 0.02X_1X_4 + 0.0004X_2X_3 + 0.0007X_2X_4 + 0.02X_3X_4$	0.9220	0.8311	0.0001	(S4)
AHPA	$Y = 673.42 - 5.13X_1 - 0.4X_2 - 4.44X_3 - 9.98X_4 + 0.03X_1^2 + 0.0001X_2^2 + 0.005X_3^2 + 0.09X_4^2 + 0.0002X_1X_2 + 0.04X_1X_3 - 0.02X_1X_4 + 0.001X_2X_3 + 0.005X_2X_4 + 0.02X_3X_4$	0.9328	0.8543	< 0.0001	(S5)

Table S2. Maximum predicted responses and optimum extraction conditions for the dependent variables.

Responses	Optimal Conditions				
	Maximum Predicted Response	*R*, mL/g (X_1)	*P*, psi (X_2)	*T*, °C (X_3)	*t*, min (X_4)
TPC (mg GAE/g dw)	25.83 ± 3.23	65	1650	150	15
TFC (mg RtE/g dw)	20.13 ± 2.69	70	1500	150	14
FRAP (μmol AAE/g dw)	131.13 ± 14.49	70	1640	150	15
DPPH (μmol AAE/g dw)	143.21 ± 17.81	60	1640	150	22
AHPA (μmol AAE/g dw)	253.24 ± 31.50	70	1630	150	16

TPC: Total polyphenol content; TFC: Total flavonoid content; FRAP: Ferric reducing antioxidant power; DPPH: 2,2-Diphenyl-1-picrylhydrazyl; AHPA: Anti-Hydrogen Peroxide Activity.

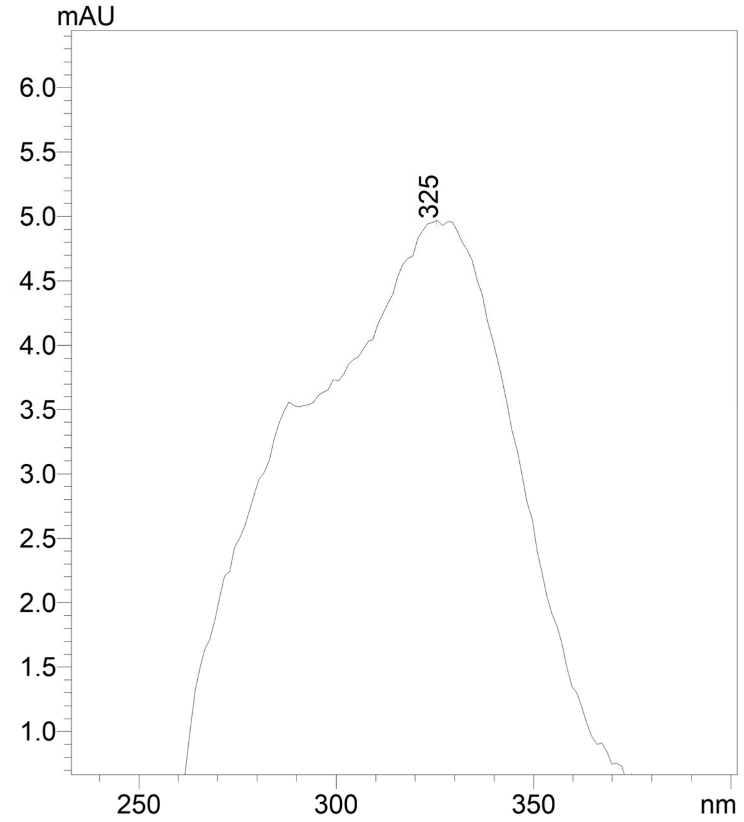

Figure S1. Ultraviolet spectra of unknown compound at retention time 22.91 min, according to the HPLC chromatograph.

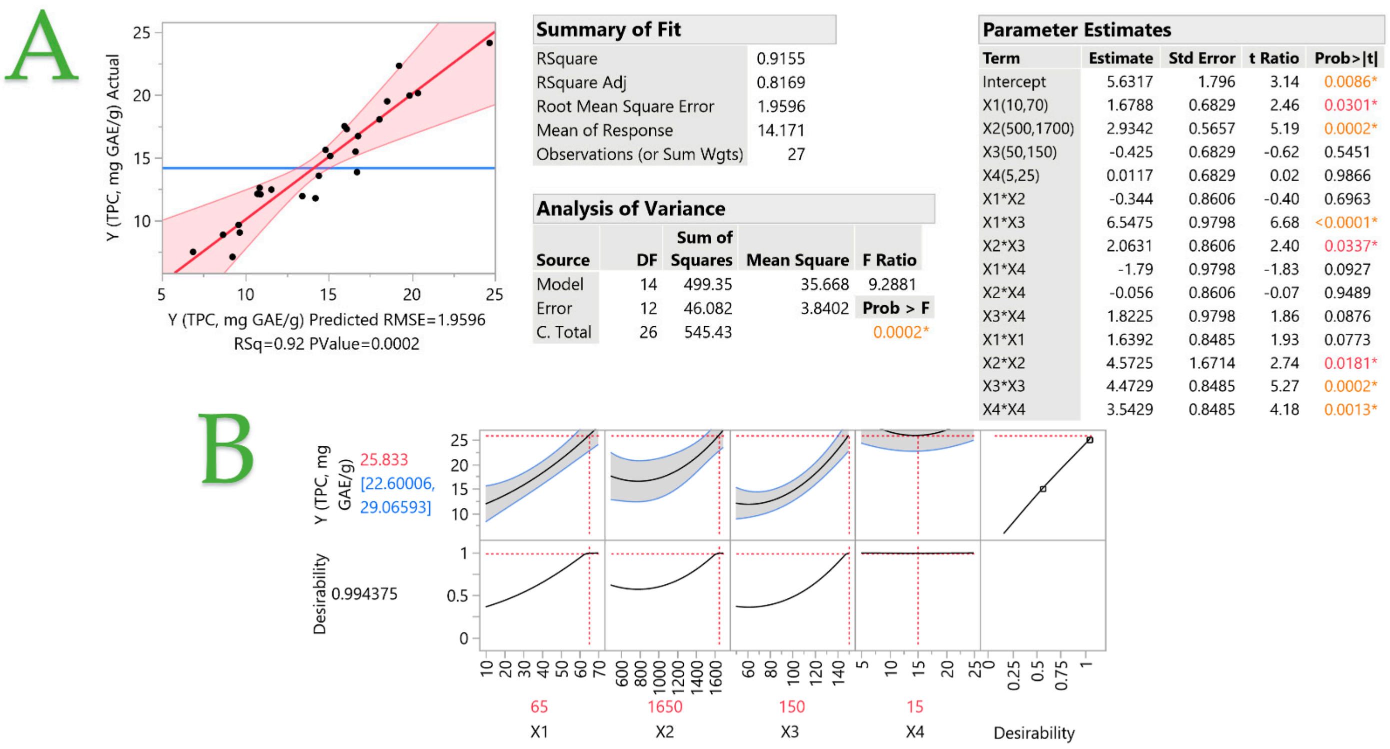

Summary of Fit

RSquare	0.9155
RSquare Adj	0.8169
Root Mean Square Error	1.9596
Mean of Response	14.171
Observations (or Sum Wgts)	27

Analysis of Variance

Source	DF	Sum of Squares	Mean Square	F Ratio
Model	14	499.35	35.668	9.2881
Error	12	46.082	3.8402	Prob > F
C. Total	26	545.43		0.0002*

Parameter Estimates

Term	Estimate	Std Error	t Ratio	Prob>|t|
Intercept	5.6317	1.796	3.14	0.0086*
X1(10,70)	1.6788	0.6829	2.46	0.0301*
X2(500,1700)	2.9342	0.5657	5.19	0.0002*
X3(50,150)	-0.425	0.6829	-0.62	0.5451
X4(5,25)	0.0117	0.6829	0.02	0.9866
X1*X2	-0.344	0.8606	-0.40	0.6963
X1*X3	6.5475	0.9798	6.68	<0.0001*
X2*X3	2.0631	0.8606	2.40	0.0337*
X1*X4	-1.79	0.9798	-1.83	0.0927
X2*X4	-0.056	0.8606	-0.07	0.9489
X3*X4	1.8225	0.9798	1.86	0.0876
X1*X1	1.6392	0.8485	1.93	0.0773
X2*X2	4.5725	1.6714	2.74	0.0181*
X3*X3	4.4729	0.8485	5.27	0.0002*
X4*X4	3.5429	0.8485	4.18	0.0013*

Figure S2. Plot **A** displays the actual response versus the predicted response (Total polyphenol content – TPC, mg GAE/g) for the optimization of *M. oleifera* extracts using different PLE extraction parameters, and plot **B** displays the desirability function. Asterisks and colored values denote statistically significant values, while inset tables include statistics relevant to the evaluation of the resulting model.

A

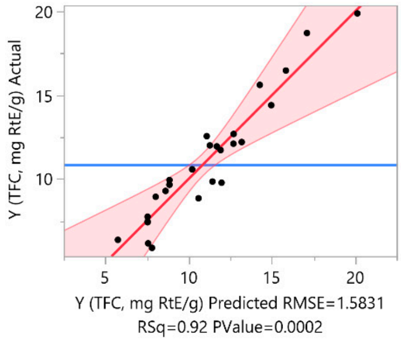

Summary of Fit	
RSquare	0.9189
RSquare Adj	0.8242
Root Mean Square Error	1.5831
Mean of Response	10.868
Observations (or Sum Wgts)	27

Analysis of Variance

Source	DF	Sum of Squares	Mean Square	F Ratio
Model	14	340.69	24.335	9.7096
Error	12	30.075	2.5062	Prob > F
C. Total	26	370.76		0.0002*

Parameter Estimates

Term	Estimate	Std Error	t Ratio	Prob>|t|
Intercept	4.8637	1.4509	3.35	0.0058*
X1(10,70)	1.4519	0.5517	2.63	0.0219*
X2(500,1700)	2.1325	0.457	4.67	0.0005*
X3(50,150)	0.4892	0.5517	0.89	0.3926
X4(5,25)	0.1538	0.5517	0.28	0.7852
X1*X2	-0.921	0.6952	-1.33	0.2098
X1*X3	5.4425	0.7916	6.88	<0.0001*
X2*X3	1.8861	0.6952	2.71	0.0189*
X1*X4	-1.083	0.7916	-1.37	0.1965
X2*X4	0.4509	0.6952	0.65	0.5289
X3*X4	0.96	0.7916	1.21	0.2485
X1*X1	0.9233	0.6855	1.35	0.2029
X2*X2	2.8305	1.3503	2.10	0.0579
X3*X3	3.6433	0.6855	5.31	0.0002*
X4*X4	2.7633	0.6855	4.03	0.0017*

B

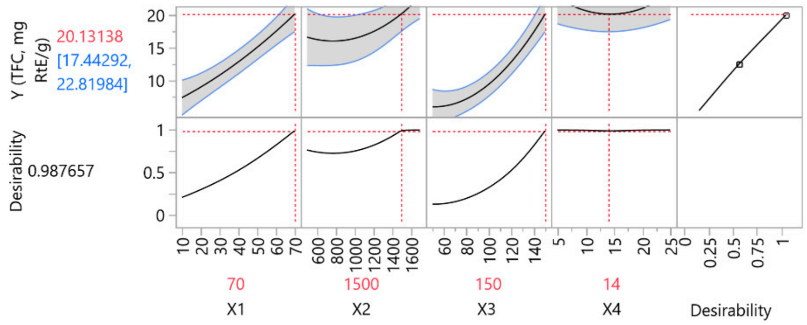

Figure S3. Plot **A** displays the actual response versus the predicted response (Total flavonoid content – TFC, mg RtE/g) for the optimization of *M. oleifera* extracts using different PLE extraction parameters, and parameters, and plot **B** displays the desirability function. Asterisks and colored values denote statistically significant values, while inset tables include statistics relevant to the evaluation of the resulting model.

A

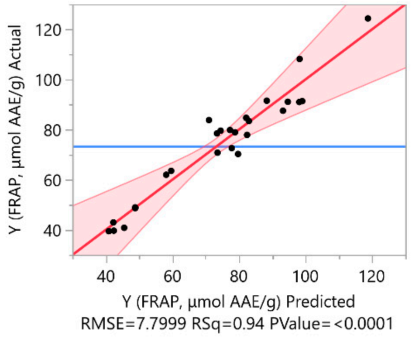

Summary of Fit	
RSquare	0.9396
RSquare Adj	0.869
Root Mean Square Error	7.7999
Mean of Response	73.324
Observations (or Sum Wgts)	27

Analysis of Variance

Source	DF	Sum of Squares	Mean Square	F Ratio
Model	14	11,348.94	810.64	13.324
Error	12	730.07	60.839	Prob > F
C. Total	26	12,079.01		<0.0001*

Parameter Estimates

Term	Estimate	Std Error	t Ratio	Prob>|t|
Intercept	30.758	7.1487	4.30	0.0010*
X1(10,70)	10.557	2.718	3.88	0.0022*
X2(500,1700)	10.06	2.2516	4.47	0.0008*
X3(50,150)	-2.663	2.718	-0.98	0.3466
X4(5,25)	7.4468	2.718	2.74	0.0179*
X1*X2	-8.388	3.4254	-2.45	0.0307*
X1*X3	33.243	3.9	8.52	<0.0001*
X2*X3	11.339	3.4254	3.31	0.0062*
X1*X4	-12.57	3.9	-3.22	0.0073*
X2*X4	0.0204	3.4254	0.01	0.9954
X3*X4	2.94	3.9	0.75	0.4655
X1*X1	13.54	3.3775	4.01	0.0017*
X2*X2	25.646	6.6528	3.85	0.0023*
X3*X3	13.214	3.3775	3.91	0.0021*
X4*X4	20.742	3.3775	6.14	<0.0001*

B

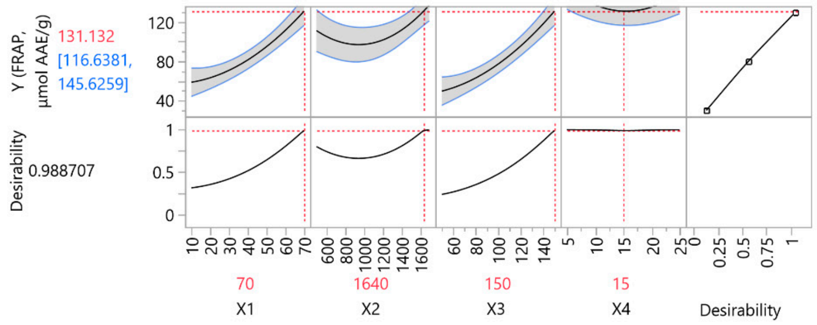

Figure S4. Plot **A** displays the actual response versus the predicted response (FRAP, µmol AAE/g) for the optimization of *M. oleifera* extracts using different PLE extraction parameters, and plot **B** displays the desirability function. Asterisks and colored values denote statistically significant values, while inset tables include statistics relevant to the evaluation of the resulting model.

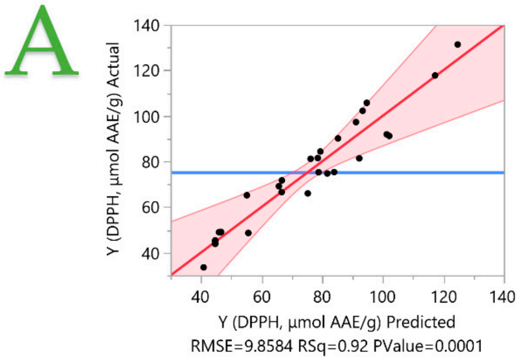

Summary of Fit	
RSquare	0.922
RSquare Adj	0.8311
Root Mean Square Error	9.8584
Mean of Response	75.333
Observations (or Sum Wgts)	27

Analysis of Variance

Source	DF	Sum of Squares	Mean Square	F Ratio
Model	14	13,795.15	985.368	10.139
Error	12	1,166.26	97.188	Prob > F
C. Total	26	14,961.41		0.0001*

Parameter Estimates

Term	Estimate	Std Error	t Ratio	Prob>|t|
Intercept	26.755	9.0354	2.96	0.0119*
X1(10,70)	7.9554	3.4353	2.32	0.0391*
X2(500,1700)	8.4167	2.8459	2.96	0.0120*
X3(50,150)	-3.038	3.4353	-0.88	0.3939
X4(5,25)	8.8829	3.4353	2.59	0.0238*
X1*X2	-1.572	4.3294	-0.36	0.7228
X1*X3	34.988	4.9292	7.10	<0.0001*
X2*X3	11.124	4.3294	2.57	0.0246*
X1*X4	-6.36	4.9292	-1.29	0.2213
X2*X4	4.1353	4.3294	0.96	0.3583
X3*X4	7.5775	4.9292	1.54	0.1502
X1*X1	10.048	4.2688	2.35	0.0365*
X2*X2	27.827	8.4086	3.31	0.0062*
X3*X3	23.66	4.2688	5.54	0.0001*
X4*X4	25.292	4.2688	5.92	<0.0001*

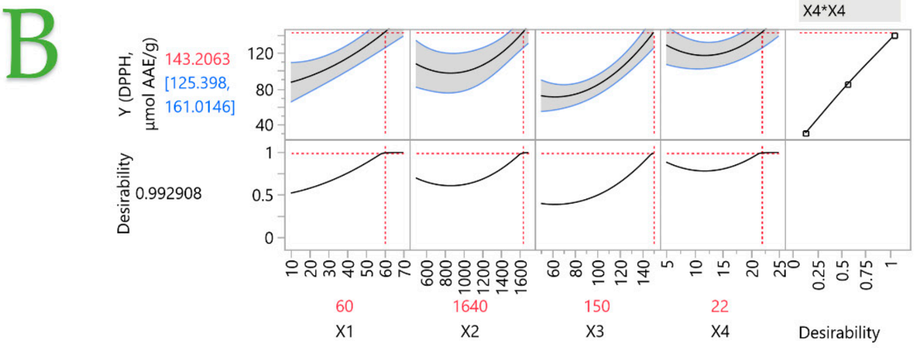

Figure S5. Plot **A** displays the actual response versus the predicted response (DPPH, µmol AAE/g) for the optimization of *M. oleifera* extracts using different PLE extraction parameters, and plot **B** displays the desirability function. Asterisks and colored values denote statistically significant values, while inset tables include statistics relevant to the evaluation of the resulting model.

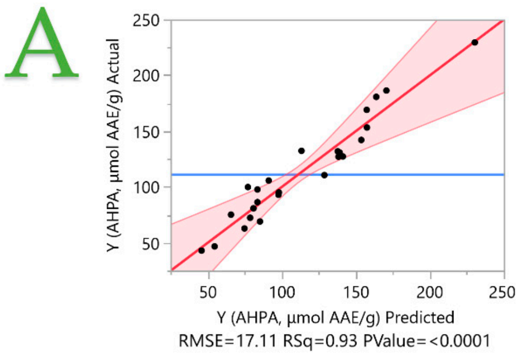

Summary of Fit	
RSquare	0.9328
RSquare Adj	0.8543
Root Mean Square Error	17.11
Mean of Response	111.56
Observations (or Sum Wgts)	27

Analysis of Variance

Source	DF	Sum of Squares	Mean Square	F Ratio
Model	14	48,724.8	3480.34	11.8888
Error	12	3,512.9	292.74	Prob > F
C. Total	26	52,237.6		<0.0001*

Parameter Estimates

Term	Estimate	Std Error	t Ratio	Prob>|t|
Intercept	66.888	15.681	4.27	0.0011*
X1(10,70)	33.077	5.9621	5.55	0.0001*
X2(500,1700)	-0.093	4.9391	-0.02	0.9854
X3(50,150)	-9.009	5.9621	-1.51	0.1566
X4(5,25)	1.8219	5.9621	0.31	0.7652
X1*X2	3.2113	7.5138	0.43	0.6767
X1*X3	56.955	8.5548	6.66	<0.0001*
X2*X3	36.779	7.5138	4.89	0.0004*
X1*X4	-6.578	8.5548	-0.77	0.4568
X2*X4	31.372	7.5138	4.18	0.0013*
X3*X4	12.29	8.5548	1.44	0.1764
X1*X1	28.857	7.4087	3.90	0.0021*
X2*X2	31.861	14.593	2.18	0.0496*
X3*X3	12.748	7.4087	1.72	0.1110
X4*X4	9.4308	7.4087	1.27	0.2271

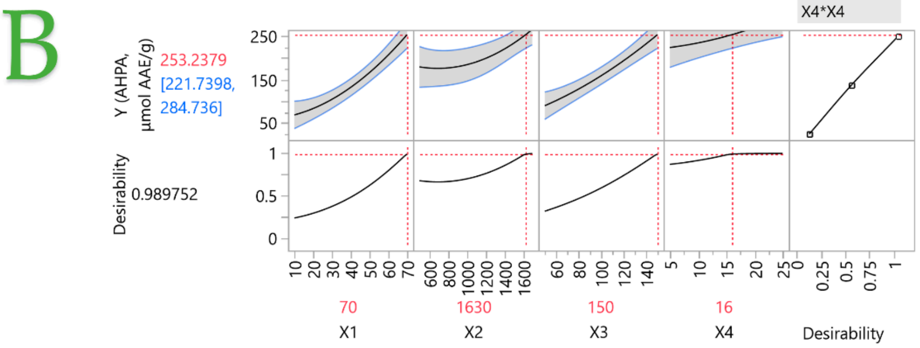

Figure S6. Plot **A** displays the actual response versus the predicted response (Anti-Hydrogen Peroxide Activity – AHPA, µmol AAE/g) for the optimization of *M. oleifera* extracts using different PLE extraction parameters, and plot **B** displays the desirability function. Asterisks and colored values denote statistically significant values, while inset tables include statistics relevant to the evaluation of the resulting model.

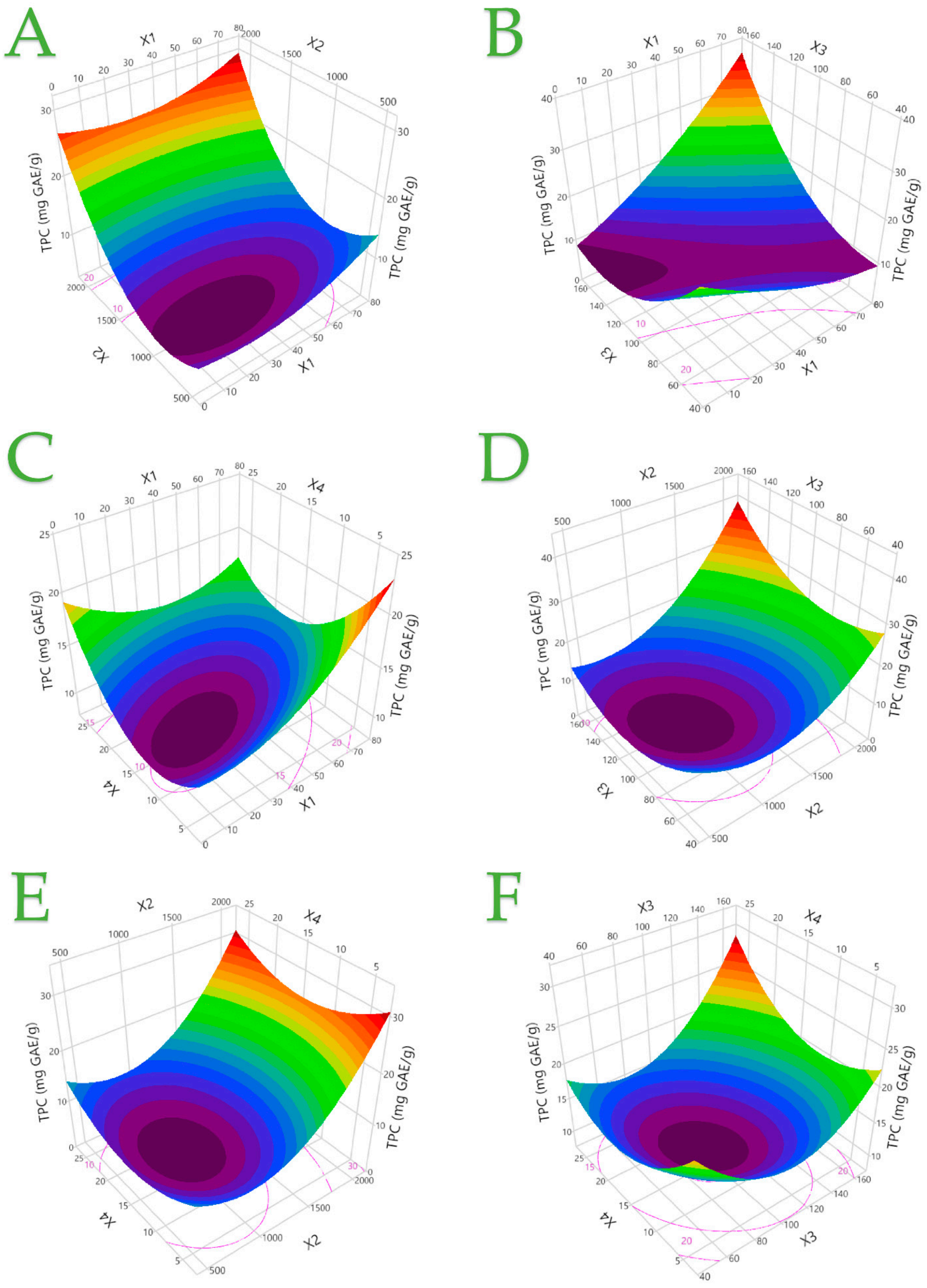

Figure S7. The optimal extraction of *M. oleifera* extracts is illustrated in 3D graphs that show the impact of the process variables considered in the response (Total polyphenol content – TPC, mg GAE/g). Plot (**A**), covariation of X_1 and X_2; plot (**B**), covariation of X_1 and X_3; plot (**C**), covariation of X_1 and X_4; plot (**D**), covariation of X_2 and X_3; plot (**E**), covariation of X_2 and X_4; plot (**F**), covariation of X_3 and X_4.

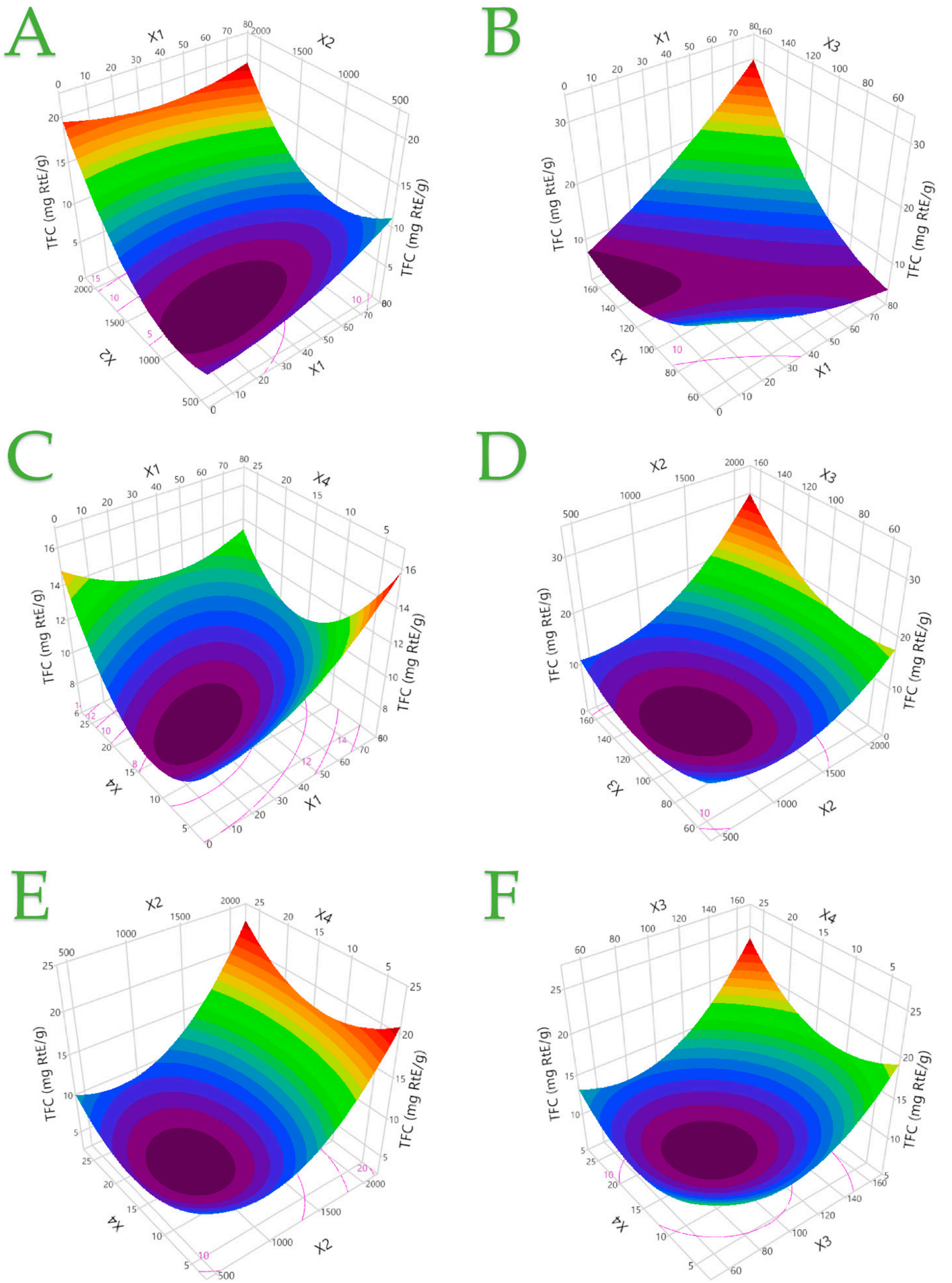

Figure S8. The optimal extraction of *M. oleifera* extracts is shown in 3D graphs that show the impact of the process variables considered in the response (Total flavonoid content – TFC, mg RtE/g). Plot (**A**), covariation of X_1 and X_2; plot (**B**), covariation of X_1 and X_3; plot (**C**), covariation of X_1 and X_4; plot (**D**), covariation of X_2 and X_3; plot (**E**), covariation of X_2 and X_4; plot (**F**), covariation of X_3 and X_4.

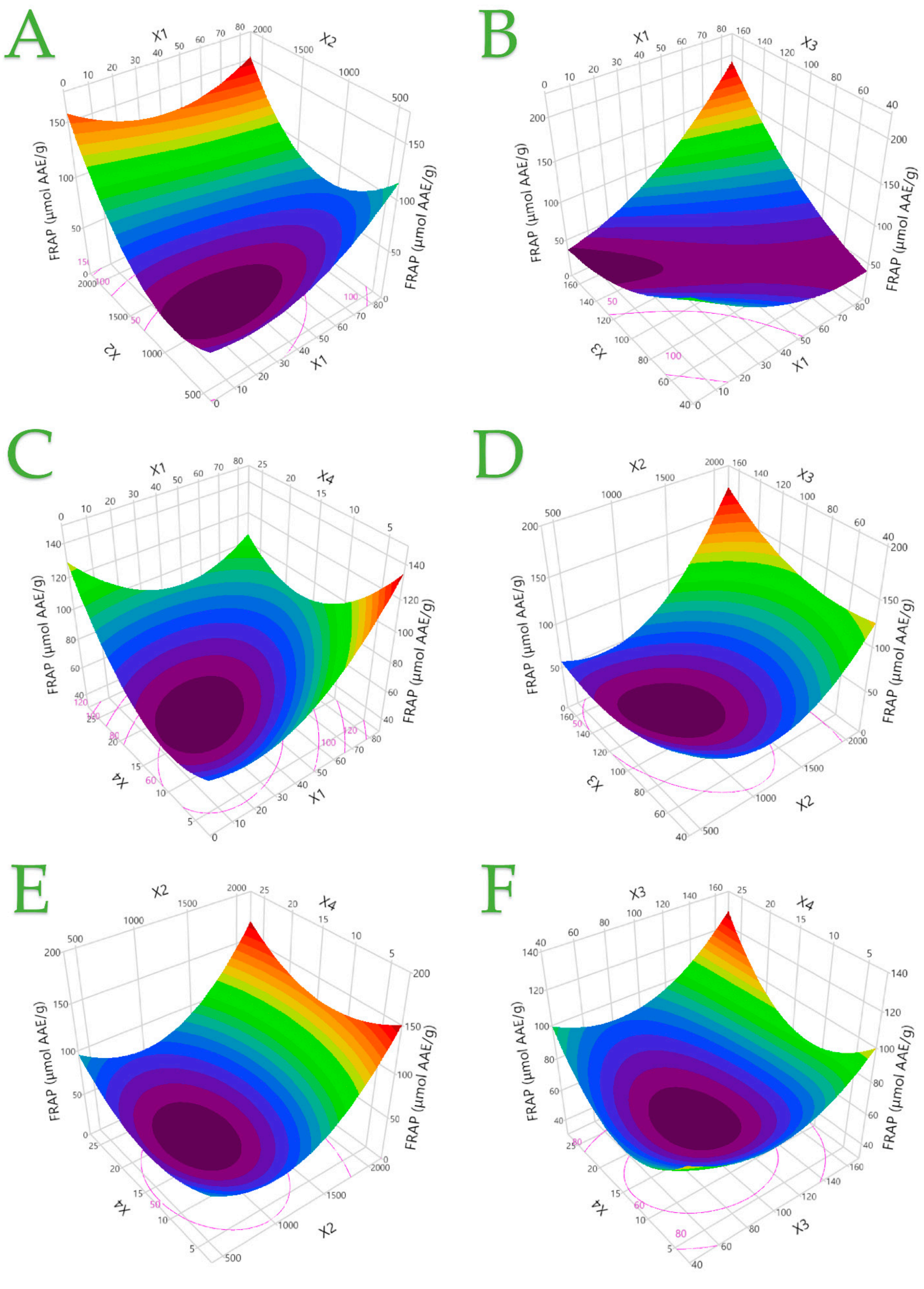

Figure S9. The optimal extraction of *M. oleifera* extracts is shown in 3D graphs that show the impact of the process variables considered in the response (FRAP, μmol AAE/g). Plot (**A**), covariation of X_1 and X_2; plot (**B**), covariation of X_1 and X_3; plot (**C**), covariation of X_1 and X_4; plot (**D**), covariation of X_2 and X_3; plot (**E**), covariation of X_2 and X_4; plot (**F**), covariation of X_3 and X_4.

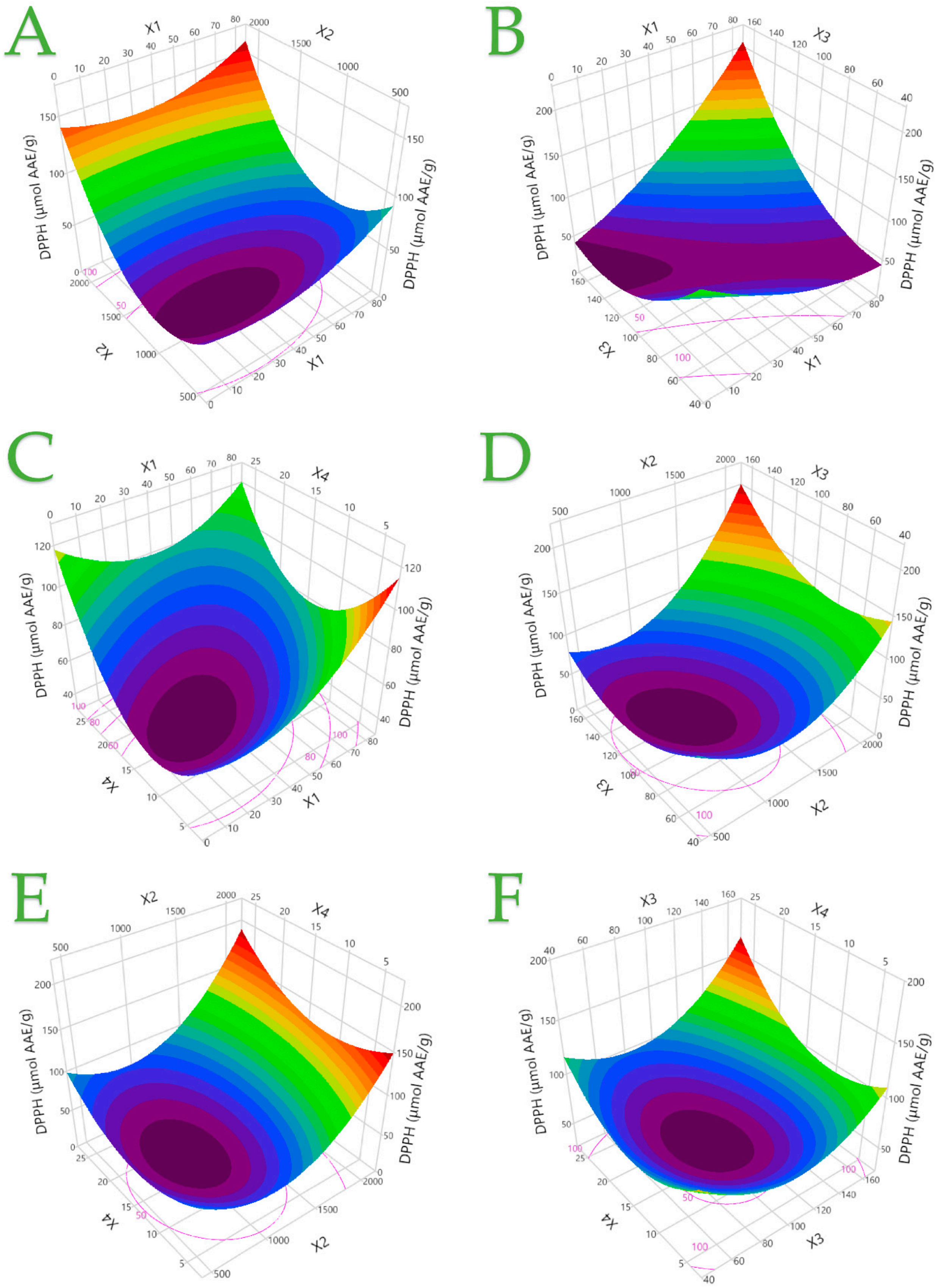

Figure S10. The optimal extraction of *M. oleifera* extracts is shown in 3D graphs that show the impact of the process variables considered in the response (DPPH, µmol AAE/g). Plot (**A**), covariation of X_1 and X_2; plot (**B**), covariation of X_1 and X_3; plot (**C**), covariation of X_1 and X_4; plot (**D**), covariation of X_2 and X_3; plot (**E**), covariation of X_2 and X_4; plot (**F**), covariation of X_3 and X_4.

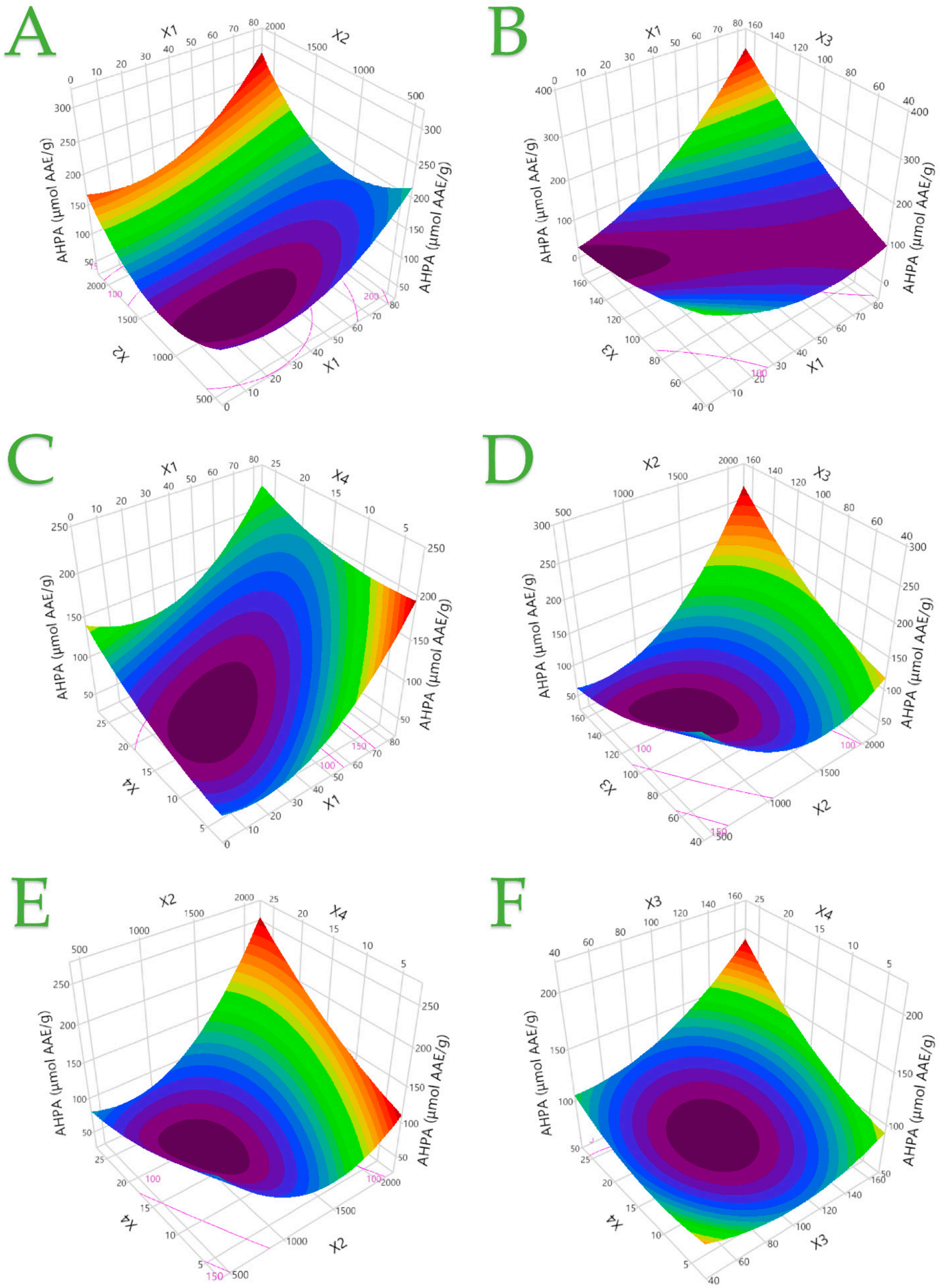

Figure S11. The optimal extraction of *M. oleifera* extracts is shown in 3D graphs that show the impact of the process variables considered in the response (Anti-Hydrogen Peroxide Activity – AHPA, µmol AAE/g). Plot (**A**), covariation of X_1 and X_2; plot (**B**), covariation of X_1 and X_3; plot (**C**), covariation of X_1 and X_4; plot (**D**), covariation of X_2 and X_3; plot (**E**), covariation of X_2 and X_4; plot (**F**), covariation of X_3 and X_4.

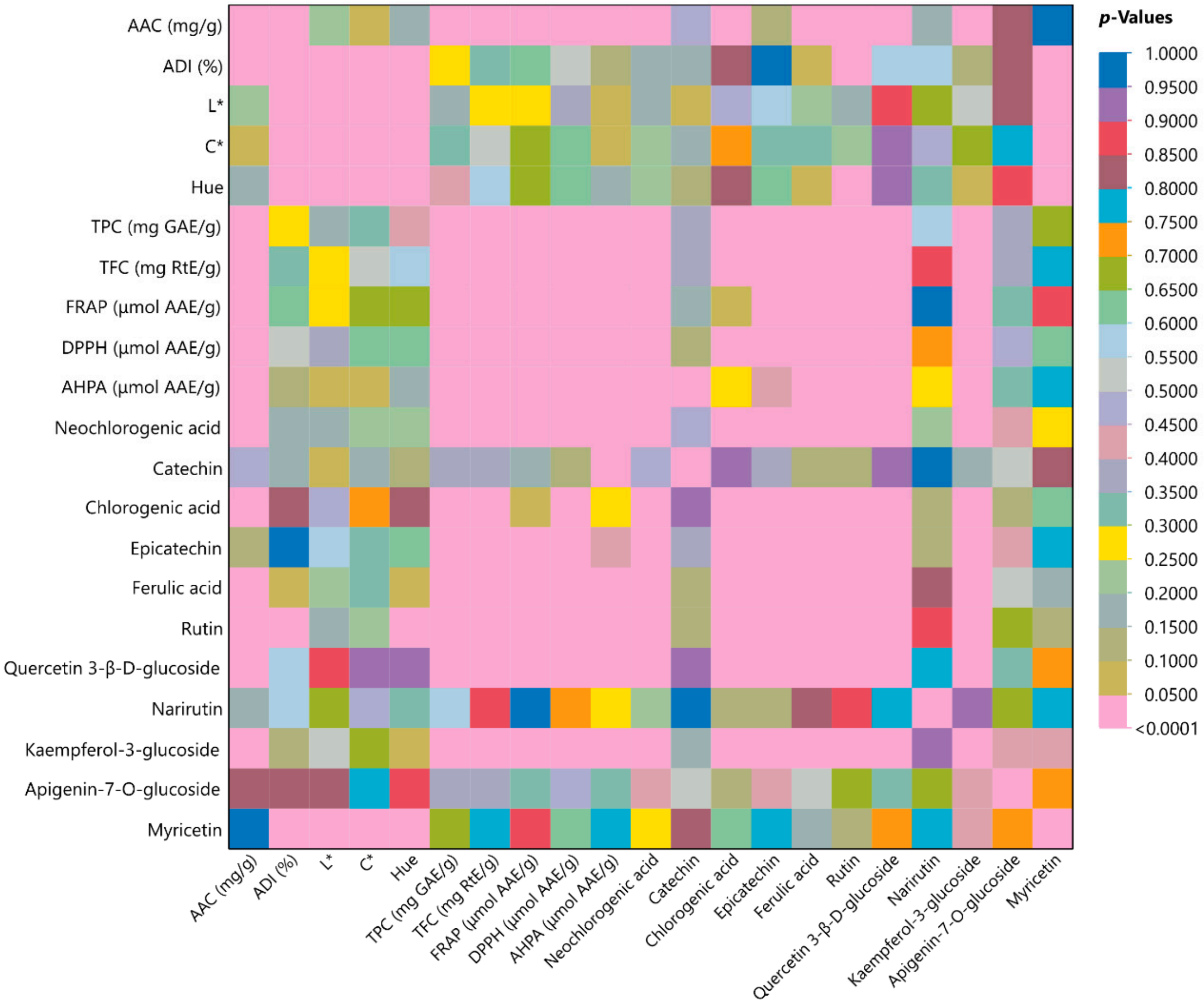

Figure S12. Multivariate color map to represent *p*-values of measured variables.
